# Phytochemical Composition and Overall Taste Modulation in Lettuce: Combination of Cultivar and Biofertiliser

**DOI:** 10.3390/plants14243864

**Published:** 2025-12-18

**Authors:** Milica Stojanović, Dragica Milosavljević, Abigaël Delcourt, Jean-Louis Hilbert, Philippe Hance, Vuk Maksimović, Jelena Dragišić Maksimović

**Affiliations:** 1 Institute for Multidisciplinary Research, Department of Life Sciences, National Institute of the Republic of Serbia, University of Belgrade, Kneza Višeslava 1, 11030 Belgrade, Serbia; dragicar@imsi.bg.ac.rs (D.M.); maxivuk@imsi.bg.ac.rs (V.M.); draxy@imsi.bg.ac.rs (J.D.M.); 2 UMRT 1158 BioEcoAgro, University of Lille, INRAE, University of Liège, University of Picardie Jules-Verne, YNCREA, University of Artois, University of Littoral Côte d’Opale, ICV-Institut Charles Viollette, Cité Scientifique, F-59000 Lille, France; delcourt.abigael@univ-lille.fr (A.D.); jean-louis.hilbert@univ-lille.fr (J.-L.H.); philippe.hance@univ-lille.fr (P.H.); 3 Joint Laboratory University of Lille-Florimond-Desprez CHIC41Health, Cité Scientifique, F-59655 Villeneuve d’Ascq, France

**Keywords:** *Lactuca sativa*, microbial fertilisers, sugars, organic acids, phenolic acids, flavonoids, sesquiterpene lactones, total sweetness index, overall taste, HPLC/UPLC/DAD

## Abstract

This study assessed the impact of biofertilisers on primary and secondary (specialised) metabolites in six lettuce cultivars (‘Kiribati’, ‘Murai’, ‘Aquino’, ‘Gaugin’, ‘Aleppo’, and ‘Carmesi’) grown in anthropogenic soil during an autumn greenhouse experiment. Four treatments were tested: control (no fertilisation), effective microorganisms (EM), *Trichoderma*, and their combination. Red cultivars showed higher total antioxidant capacity (TAC) and total phenolic content (TPC), with red Lollo ‘Carmesi’ having the highest TAC, TPC, carotenoids, total soluble solids, sweetness index, and sugars. Red Oak ‘Murai’ exhibited the highest chlorophyll b and total chlorophyll, while green cultivars ‘Kiribati’ and ‘Aquino’ excelled in chlorophyll a and overall taste, respectively. Biofertilisers did not affect TAC or most chlorophyll types but increased TPC (EM by 18.6% and combined treatment by 19.6%) and chlorophyll a (EM by 28.6% and *Trichoderma* by 23.8%). Combined fertilisers improved taste with reduced glucose and fructose content and sweetness index, though sucrose remained unchanged compared to the control. Major organic acids (malic, citric, and tartaric) were most abundant in ‘Murai’ and ‘Kiribati’, unaffected by treatments. Phenolics content peaked in ‘Murai’ and ‘Carmesi’, characterised by chicoric and chlorogenic acid, caffeoylquinic acid glucoside, and flavonoids (quercetin derivatives, kaempferol); biofertilisers mainly influenced gallic acid, while kaempferol was affected by all biofertilisers and increased in the range of 12.5–25%. The key sesquiterpene lactones identified were lactucin, lactucopicrin-15-oxalate, and 11β,13-dihydrolactucin. The content of sesquiterpene lactones analysed in this study increased significantly, especially with EM treatment (14.7–185.7%) and combined fertilisers (12.5–128.6%), highlighting the lactone-rich cultivars ‘Carmesi’ and ‘Gaugin’. Red cultivars ‘Carmesi’ and ‘Murai’ exhibited the most favourable phytochemical profiles, suitable for cultivation and processing of quality-based products. In contrast, the green cultivar ‘Aquino’ received the highest sensory scores, delivering the most appealing overall taste despite its lower metabolite content. EM treatment and combined fertilisers are recommended for increasing chlorophyll a, myo-inositol, TSS, propionic acid, TPC, kaempferol, and major lactones under greenhouse autumn conditions.

## 1. Introduction

Lettuce (*Lactuca sativa* L.), an annual leafy vegetable in the Asteraceae family, is one of the most consumed and widely cultivated salad crops worldwide, with a combined global production of lettuce and chicory exceeding 28 million metric tonnes in 2023 [1]. Consumer preferences are shaped by attributes like colour, size, texture, and taste, which also influence market prices [2]. This heightened interest has focused attention on lettuce’s rich phytochemical profile, which contributes to both nutritional value and sensory characteristics [3]. Lettuce is a cool-season crop with moderate temperature requirements and a relatively short growing period. It is typically cultivated during autumn, winter, and spring [4], although year-round production is possible through advanced cultivation methods. Lettuce can be grown via multiple production systems, including open-field cultivation, controlled environments, and hydroponics.

Lettuce is a rich source of beneficial phytochemicals, including vitamins, minerals, carotenoids, chlorophyll, polyphenols, and sesquiterpene lactones [5,6]. It is low in calories, comprising about 95% water. Compared to green cultivars, red lettuce varieties exhibit significantly higher total phenolic content and antioxidant activity [7,8]. Epidemiological studies and meta-analyses suggest that long-term consumption of diets rich in plant-derived polyphenols and antioxidants provides protection against diseases related to metabolic syndrome, various cancers, osteoporosis, and neurodegenerative disorders [9].

Besides their benefits for human health, plant metabolites perform diverse vital functions within plants themselves. Primary metabolites in plants (sugars, organic acids) and main pigments, such as chlorophyll and carotenoids, are essential compounds directly involved in fundamental physiological processes, including energy production, growth, development, and photosynthesis. Sugars provide energy and carbon skeletons [10], organic acids participate in metabolic pathways and pH regulation [11], chlorophyll captures light energy for photosynthesis, and carotenoids assist in light harvesting and protect against photooxidative damage [12]. Specialised metabolites, including lactones and phenolics, perform distinct ecological functions, like defence against herbivores and pathogens, attraction of pollinators, and tolerance to environmental stresses [13,14]. Phenolic compounds specifically help plants to combat abiotic and biotic stresses by providing antioxidant protection, shielding from ultraviolet radiation, enhancing nutrient availability in the soil, and mitigating heavy metal toxicity and salt stress [15]. Thus, while primary metabolites are indispensable for sustaining vital physiological and biochemical functions in plants, specialised metabolites enable plants to adapt, survive, and interact within their environment, playing crucial roles in defence mechanisms and stress resilience.

Taste in lettuce is a complex trait influenced by multiple metabolites [16,17]. Sugars, such as glucose, fructose, and sucrose, contribute to sweetness, which balances bitterness and strongly affects consumer preference. Organic acids and phenolic compounds contribute to bitterness, sourness, and astringency, with phenolics playing a lesser but significant role compared to sesquiterpene lactones. Sesquiterpene lactones are key contributors to lettuce’s bitter taste beyond their ecological functions. Importantly, taste perception depends more on the balance between bitter and sweet components than on the concentration of individual metabolites [18].

Despite technological advances, recent data show that 95% of global food is still grown on soil, emphasising the critical need for sustainable soil management [19]. Increasing soil degradation, pollution, and climatic shifts, which reduce arable land, have intensified fears of global food shortages and growing food insecurity [20]. Anthropogenic soils, considered created or modified through human activities, are prevalent in agricultural and urban areas [21]. The inclusion of anthropogenic soils that are free from heavy metal contamination, toxic compounds, and other pollutants, combined with sustainable management practices such as biofertiliser use, offers a promising opportunity to expand areas available for plant cultivation.

Chemical fertilisation is widely employed in conventional agriculture to boost yields, but its excessive use can lead to environmental issues, including soil degradation, nutrient imbalances, and water pollution [22]. Consequently, the application of more eco-friendly fertilisers to replace chemical ones has become a major agricultural challenge in recent years [23]. Biofertilisers contain beneficial microorganisms, active or dormant, that colonise the rhizosphere or internal plant tissues. These microbes enhance nutrient availability, especially nitrogen, phosphorus, and potassium, improve nutrient mobilisation, stimulate root growth, and ultimately boost crop productivity [24]. Among these, the genus *Trichoderma* is well-studied for its plant growth-promoting effects. It acts both as a biological control agent against pathogens and as a biostimulant [25]. *Trichoderma* chemically interacts with plant roots by secreting auxins, small peptides, volatile organic compounds, and other bioactive metabolites [26]. This colonisation induces root branching, enhances nutrient uptake, promotes growth and yield, and strengthens plant defences against abiotic and biotic stresses [26,27]. Previous research has shown positive effects of beneficial microorganisms and biofertilisers on lettuce quality traits [28,29,30].

However, gaps remain regarding biofertiliser impacts on primary and specialised metabolites, particularly in anthropogenic soils or soils with low to medium organic matter content under controlled environments, limiting understanding of plant biochemical responses. Soil remains the primary medium for plant cultivation globally, and expanding the use of anthropogenic soils with low to moderate organic matter content is essential for increasing arable land area, enhancing food security, and supporting sustainable vegetable production. Although challenging, these soils provide valuable opportunities to meet the rising demand for food production. This experiment focuses on the main effects of lettuce cultivar and biofertiliser application on primary and specialised metabolite contents and sensory attributes, which are crucial determinants of lettuce quality, affecting consumer acceptance and market value. Given the variability in metabolic profiles and taste among lettuce cultivars, evaluating their individual effects alongside those of biofertilisers provides important insights for optimising quality. Understanding how biofertilisers independently influence metabolite synthesis and taste in these soils reveals underlying biochemical mechanisms that contribute to improved plant quality.

Several hypotheses are proposed to address the research questions in our study. First, cultivar and biofertiliser are two primary factors that independently influence metabolite concentrations and sensory taste attributes (i). Application of biofertilisers is expected to elevate primary metabolite levels and enhance specialised metabolite accumulation (ii). Furthermore, combined fertiliser treatment is anticipated to produce a synergistic effect, enhancing metabolite levels more than biofertilisers alone or untreated control (iii). Ultimately, biofertiliser use is likely to improve overall taste pleasantness (iv).

Thus, this study aims to evaluate the contributions of lettuce cultivars grown in anthropogenic soil with moderate organic matter content and the effects of biofertiliser applications on quality-related metabolites and overall taste. The goal is to provide evidence-based recommendations for selecting optimal cultivar and biofertiliser combinations tailored to low–moderate organic matter soils, benefiting growers, the processing industry, consumers, and other stakeholders focused on improving product quality.

## 2. Results

### 2.1. Spectrophotometric Assays

The results of TAC measurements are presented in Table 1. TAC was significantly influenced by cultivar, whereas biofertiliser treatments had no significant effect. The red cultivar ‘Carmesi’ exhibited the highest TAC value (0.52 mg AA eq g^−1^ FW), while the lowest was recorded in the green cultivar ‘Kiribati’ (0.24 mg AA eq g^−1^ FW). Overall, red cultivars demonstrated higher TAC levels compared to their green counterparts.

Both cultivar and fertiliser had significant effects on TPC (Table 1). Consistent with TAC, all red cultivars contained significantly higher phenolic levels than green cultivars. The red cultivar ‘Carmesi’ exhibited the highest TPC (365.66 µg GA eq g^−1^ FW), whereas the green cultivar ‘Aquino’ had the lowest (127.11 µg GA eq g^−1^ FW). Compared to the control, application of EM Aktiv and combined fertilisers significantly increased TPC by 18.6% and 19.6%, respectively.

Cultivar had a significant effect on chlorophyll levels (Table 1). Within the Oak lettuce cultivars, ‘Kiribati’ exhibited the highest chlorophyll a content (3.1 µg g^−1^ FW), while ‘Murai’ showed the greatest chlorophyll b (4.2 µg g^−1^ FW) and total chlorophyll (a + b) (7.4 µg g^−1^ FW) concentrations. Conversely, ‘Gaugin’ displayed the lowest chlorophyll a content (1.9 µg g^−1^ FW), and ‘Aleppo’ had the lowest chlorophyll b (2.0 µg g^−1^ FW) and total chlorophyll (3.8 µg g^−1^ FW) levels. Application of biofertilisers significantly increased chlorophyll a content, with EM Aktiv and Vital Tricho enhancing levels by 28.6% and 23.8%, respectively, compared to the control. However, fertiliser treatments did not produce significant changes in chlorophyll b or total chlorophyll (a + b) content.

Regarding total carotenoids (Table 1), both cultivar and fertiliser had significant effects. The red Lollo cultivar ‘Carmesi’ exhibited the highest carotenoid content (1.8 µg g^−1^ FW), whereas the green Lollo cultivar ‘Aleppo’ showed the lowest levels (0.7 µg g^−1^ FW). Fertiliser application with Vital Tricho resulted in higher total carotenoid content compared to EM Aktiv.

### 2.2. Sugars, Total Sweetness Index (TSI), Total Soluble Solids (TSS)

The HPLC analysis of sugars is summarised in Table 2. Fructose was the most abundant sugar detected in the lettuce samples, followed by glucose, sucrose, and myo-inositol. Additionally, two unidentified sugar compounds—referred to as compound **1** (expressed in arabinose equivalents) and compound **2** (expressed in maltose equivalents)—were observed. Although their HPLC peaks are closely aligned with arabinose and maltose, their retention times do not match the standards for these sugars, suggesting that they are distinct compounds.

Cultivar and biofertiliser showed a significant influence on sugar content, excluding the effect of fertiliser on sucrose content. The red Lollo-type cultivar ‘Carmesi’ contained the highest levels of fructose (18.09 mg g^−1^ FW), glucose (15.33 mg g^−1^ FW), sucrose (8.95 mg g^−1^ FW), and unidentified compound **2** (7.41 mg maltose eq g^−1^ FW). Additionally, red cultivars ‘Murai’ and ‘Gaugin’ displayed distinctive sugar profiles: ‘Murai’ had the highest myo-inositol concentration (3.38 mg g^−1^ FW), while ‘Gaugin’ showed the highest level of unidentified compound **1** (4.41 mg arabinose eq g^−1^ FW).

Compared to the control, EM Aktiv application increased myo-inositol by 21.4%, compound **1** by 34.3%, and compound **2** by 56.9%. Vital Tricho treatment resulted in an 82.1% increase in compound **2**. In contrast, combined fertilisers reduced glucose, fructose, and myo-inositol levels by 24%, 24.6%, and 17.1%, respectively. Similarly, Vital Tricho decreased fructose content by 17.4%. Among the fertiliser treatments, EM Aktiv consistently led to significantly higher levels of glucose and compound **2** (compared to combined fertilisers), as well as fructose and myo-inositol (compared to both Vital Tricho and combined fertilisers).

Total sweetness index (TSI) and total soluble solids (TSS) were significantly impacted by cultivar and fertiliser (Table 2). The red cultivar ‘Carmesi’ exhibited the highest TSI (47.74) and TSS (4.17 °Brix), while the lowest values were recorded in cultivars ‘Kiribati’ (TSI: 30.15) and ‘Aleppo’ (TSS: 3.04 °Brix), respectively. Interestingly, biofertiliser application produced contrasting effects on TSS and TSI: combined fertilisers significantly increased TSS by 26.6% relative to the control, whereas the same combination reduced TSI by 22.2%. Consistent with sugar content results, EM Aktiv also significantly elevated TSI compared to Vital Tricho and combined fertiliser treatments.

### 2.3. Organic Acids

The HPLC analysis of organic acids is summarised in Table 3. Malic acid was the most abundant organic acid detected in the lettuce samples, followed by propionic, citric, and tartaric acids. Additionally, two unidentified organic acids—referred to as compound **3** (expressed in mg formic acid equivalents) and compound **4** (expressed in mg oxalic acid equivalents)—were observed. Their HPLC peaks are closely aligned with formic and oxalic acids, respectively; however, their retention times do not match those of the standard compounds, indicating that they are distinct.

Cultivar had a significant effect on organic acid content (Table 3). The red cultivar ‘Murai’ showed the highest concentrations of malic acid (4.05 mg g^−1^ FW) and citric acid (0.22 mg g^−1^ FW). Propionic acid peaked in the red cultivar ‘Gaugin’ (1.44 mg g^−1^ FW). Among green cultivars, ‘Aleppo’ had the highest level of the unidentified compound **3** (0.18 mg formic acid eq g^−1^ FW), while ‘Aquino’ exhibited the highest level of compound **4** (0.05 mg oxalic acid eq g^−1^ FW). The green cultivar ‘Kiribati’ contained the highest amounts of tartaric acid (0.14 mg g^−1^ FW), fumaric acid (72.06 µg g^−1^ FW), and shikimic acid (39.58 µg g^−1^ FW).

Biofertiliser treatments did not significantly affect the majority of organic acids (malic, citric, tartaric, and fumaric acids), except for propionic acid, shikimic acid, and the two unidentified compounds. Compared to the control, application of EM Aktiv increased propionic acid by 29.7%, compound **3** by 12.5%, and compound **4** by 25%. Vital Tricho treatment led to an 18.8% decrease in compound **3**. Notably, EM Aktiv treatment resulted in significantly higher shikimic acid levels compared to Vital Tricho and the combined fertiliser application.

### 2.4. Phenolic Acids

Phenolic acid concentrations were significantly affected by cultivar (Table 4). The major phenolic acids detected in lettuce cultivars included chicoric acid, caffeoylquinic acid glucoside, gallic acid, and chlorogenic acid. The red cultivar ‘Murai’ exhibited the highest levels of chicoric acid (4.08 µg caffeic acid eq g^−1^ FW), caffeoylquinic acid glucoside (2.01 µg caffeic acid eq g^−1^ FW), gallic acid (1.71 µg g^−1^ FW), chlorogenic acid (1.51 µg g^−1^ FW), and caffeic acid glucuronide (0.77 µg caffeic acid eq g^−1^ FW). Meanwhile, the red cultivar ‘Carmesi’ showed the highest concentrations of caffeic acid-3-glucoside (0.72 µg caffeic acid eq g^−1^ FW) and *p*-coumaroylquinic acid (0.13 µg coumaric acid eq g^−1^ FW). Interestingly, the green cultivar ‘Aleppo’ had the highest level of caffeic acid (0.33 µg g^−1^ FW).

In general, red cultivars tended to accumulate greater amounts of gallic, chlorogenic, and *p*-coumaroylquinic acids compared to their green counterparts. However, patterns varied by lettuce type: for chicoric acid, green cultivars of the Lollo and Salanova types showed higher levels than their red counterparts, whereas a contrasting pattern was observed in the Oak type.

Fertiliser application did not significantly affect overall phenolic acid content, except for gallic acid. While there were no significant differences between the control and fertiliser treatments as a whole, application of EM Aktiv and the combined fertilisers tended to produce higher gallic acid levels than Vital Tricho.

### 2.5. Flavonoids

Flavonoid concentrations were significantly influenced by cultivar, while fertiliser treatment alone showed no significant effect—except in the case of kaempferol content (Table 5).

Lettuce samples contained quercetin and its derivatives, as well as kaempferol and its derivative. The predominant flavonoid was quercetin-3-O-(6″-O-malonyl)-glucoside, followed by quercetin-3-glucoside, quercetin-3′-O-glucuronide, and quercetin 3-O-(6″-malonyl-glucoside) 7-O-glucoside. Red cultivars consistently accumulated higher levels of most flavonoids compared to green counterparts, except for kaempferol. The red cultivar ‘Carmesi’ exhibited the highest concentrations of quercetin-3-O-(6″-O-malonyl)-glucoside (11.13 µg quercetin eq g^−1^ FW), quercetin-3′-O-glucuronide (4.81 µg quercetin eq g^−1^ FW), quercetin 3-O-(6″-malonyl-glucoside) 7-O-glucoside (1.96 µg quercetin eq g^−1^ FW), quercetin 3-O-(6″-acetyl-glucoside) (0.83 µg quercetin eq g^−1^ FW), and kaempferol (6-O-malonyl) glucoside (0.29 µg kaempferol eq g^−1^ FW). Meanwhile, red cultivars ‘Gaugin’ and ‘Murai’ showed the highest levels of quercetin-3-glucoside (5.62 µg quercetin eq g^−1^ FW) and kaempferol (0.11 µg g^−1^ FW), respectively. Generally, the green cultivar ‘Aquino’ exhibited the lowest levels of most flavonoids. Fertiliser application did not significantly increase flavonoid concentrations overall, except for kaempferol. Both individual fertilisers and their combination enhanced kaempferol in the range of 12.5–25%.

### 2.6. Sesquiterpene Lactones

Cultivar and biofertiliser treatments significantly influenced the peak areas of lactones, except for the effect of fertilisation on 11β,13-dihydrolactucopicrin (Table 6). The main sesquiterpene lactones detected in lettuce were lactucin, lactucopicrin-15-oxalate, 11β,13-dihydrolactucin, 11β,13-dihydrolactucin-15-glucoside, and lactucopicrin. Among cultivars, the red ‘Carmesi’ showed the highest peak areas for total lactones (6.38), lactucopicrin-15-oxalate (2.28), lactucopicrin (0.64), 8-deoxylactucin (0.44), lactucin-15-oxalate (0.29), 11β,13-dihydrolactucopicrin-15-oxalate (0.075), and 11β,13-dihydrolactucopicrin (0.05). The red cultivar ‘Gaugin’ exhibited the highest values for lactucin (1.82) and 11β,13-dihydrolactucin-15-glucoside (1.13). In contrast, 11β,13-dihydrolactucin was the predominant lactone in the green Oak cultivar ‘Kiribati’. 11β,13-dihydro-8-deoxylactucin was uniquely identified in the green ‘Aquino’ cultivar under EM Aktiv treatment (0.28 ± 0.02). Overall, red cultivars had higher lactone peak areas than their green counterparts in the Salanova and Lollo, while the green Oak cultivar surpassed the red in 11β,13-dihydrolactucin, lactucin-15-oxalate, 11β,13-dihydrolactucopicrin-15-oxalate, 11β,13-dihydrolactucin-15-glucoside, and total lactones.

The application of EM Aktiv and combined biofertilisers generally enhanced lactone peak areas. Compared to the control, EM Aktiv increased 8-deoxylactucin by 185.7%, 11β,13-dihydrolactucin by 32.7%, lactucopicrin by 25%, lactucopicrin-15-oxalate by 47.5%, and total lactones by 14.7%. Combined fertilisation increased lactucin by 32.1%, 8-deoxylactucin by 128.6%, lactucopicrin-15-oxalate by 25%, 11β,13-dihydrolactucopicrin-15-oxalate by 28%, and total lactones by 12.5%. Vital Tricho alone significantly raised lactucin levels by 26.4%. Interestingly, all fertilisation treatments led to a decrease in lactucin-15-oxalate peak areas by 63–81.5%. Regarding 11β,13-dihydrolactucin-15-glucoside, peak areas showed no significant difference between control and treated plants, although Vital Tricho induced higher peaks compared to the combined fertilisation treatment.

### 2.7. Overall Taste

Cultivar and fertiliser had a significant impact on overall taste according to the sensory analysis (Table 7). Overall taste was rated on a 1-to-5 scale, where 1 indicated very poor taste and 5 indicated very good taste. The green Salanova cultivar ‘Aquino’ received the highest overall taste score (3.08), while the green cultivar ‘Aleppo’ had the lowest score (2.54). All cultivars scored between 2 and 3, reflecting a range from poor to neutral-acceptable taste. Lower scores were mainly attributed to a bitter flavour, which negatively affected the overall taste and panellists’ perceptions. Among the fertiliser treatments, the combined fertilisers produced the most favourable taste outcomes compared to Vital Tricho, though no significant differences were found between the fertiliser treatments and the control.

### 2.8. Correlations

A heat map presenting Pearson correlation coefficients and their significance for all tested compounds is shown in Figure S1 and Table S1; the results presented here are limited to the key compounds within the primary and specialised metabolite groups.

Fructose, as the predominant sugar, exhibited very strong positive correlations with TSI (0.93**) and glucose (0.85**), as well as a moderately strong positive correlation with myo-inositol (0.46**). It also showed weak positive correlations with shikimic acid (0.33**), 8-deoxylactucin (0.32**), sucrose (0.28*), lactucopicrin-15-oxalate (0.27*), 11β,13-dihydrolactucopicrin-15-oxalate (0.27*), quercetin-3′-O-glucuronide (0.26*), lactucin-15-oxalate (0.25*), and quercetin 3-O-(6″-malonyl-glucoside) 7-O-glucoside (0.25*). Conversely, fructose showed weak negative correlations with 11β,13-dihydrolactucin-15-glucoside (−0.26*), chicoric acid (−0.25*), and lactucin (−0.24*).

Malic acid, as a major organic acid, showed moderate positive correlations with citric acid (0.53**) and fumaric acid (0.45**), as well as weak positive correlations with chlorophyll b (0.30*), shikimic acid (0.29*), chlorophyll (a + b) (0.27*), propionic acid (0.27*), tartaric acid (0.26*), and caffeoylquinic acid glucoside (0.24*). A weak negative correlation was observed between malic acid and 11β,13-dihydrolactucopicrin (−0.30*).

Chicoric acid, a predominant phenolic acid, showed moderate positive correlations with caffeoylquinic acid glucoside (0.58**) and caffeic acid glucuronide (0.51**). A weak positive correlation was observed with chlorogenic acid (0.36**), caffeic acid (0.33**), caffeic acid-3-glucoside (0.30*), kaempferol (0.30*), and chlorophyll b (0.25*). In contrast, it exhibited weak negative correlations with 11β,13-dihydrolactucin (−0.40**), 11β,13-dihydrolactucopicrin-15-oxalate (−0.36**), TSI (−0.32**), 11β,13-dihydrolactucopicrin (−0.32**), propionic acid (−0.31**), *p*-coumaroylquinic acid (−0.29*), total carotenoids (−0.29*), quercetin-3-glucoside (−0.28*), sucrose (−0.26*), TPC (−0.26*), fructose (−0.25*), 11β,13-dihydrolactucin-15-glucoside (−0.24*), and TSS (−0.23*).

Quercetin-3-O-(6″-O-malonyl)-glucoside, a major flavonoid, showed very strong positive correlations with quercetin 3-O-(6″-acetyl-glucoside) (0.97**), quercetin 3-O-(6″-malonyl-glucoside) 7-O-glucoside (0.91**), kaempferol (6-O-malonyl) glucoside (0.90**), quercetin-3-glucoside (0.89**), quercetin-3′-O-glucuronide (0.88**), and *p*-coumaroylquinic acid (0.86**). A strong positive correlation was observed with TPC (0.78**), chlorogenic acid (0.71**), TAC (0.67**), 11β,13-dihydrolactucopicrin-15-oxalate (0.67**), gallic acid (0.66**), lactucopicrin-15-oxalate (0.63**), and total lactones (0.61**). A moderate positive correlation was observed with lactucin (0.60**), total carotenoids (0.56**), 11β,13-dihydrolactucopicrin (0.54**), sucrose (0.45**), 8-deoxylactucin (0.43**), and lactucopicrin (0.41**). A weak positive correlation was observed with 11β,13-dihydrolactucin (0.33**), caffeic acid glucuronide (0.33**), TSI (0.29*), and citric acid (0.25*). In contrast, it demonstrated moderate negative correlations with tartaric acid (−0.46**) and fumaric acid (−0.46**).

Lactucin, as a major lactone, showed a strong positive correlation with total lactones (0.79**), quercetin-3-glucoside (0.65**), chlorogenic acid (0.64**), TPC (0.61**), and quercetin 3-O-(6″-acetyl-glucoside) (0.61**). A moderate positive correlation was observed with quercetin-3-O-(6″-O-malonyl)-glucoside (0.60**), 11β,13-dihydrolactucin (0.58**), *p*-coumaroylquinic acid (0.55**), quercetin-3′-O-glucuronide (0.52**), kaempferol(6-O-malonyl) glucoside (0.52**), quercetin 3-O-(6″-malonyl-glucoside) 7-O-glucoside (0.50**), lactucopicrin-15-oxalate (0.48**), 11β,13-dihydrolactucopicrin (0.46**), citric acid (0.44**), and gallic acid (0.43**). A weak positive correlation was observed with 11β,13-dihydrolactucopicrin-15-oxalate (0.40**), caffeic acid glucuronide (0.38**), caffeoylquinic acid glucoside (0.38**), TAC (0.35**), lactucopicrin (0.34**), 11β,13-dihydrolactucin-15-glucoside (0.33**), chlorophyll (a + b) (0.33**), total carotenoids (0.29*), chlorophyll b (0.24*), and propionic acid (0.24*). A weak negative correlation was found with fructose (−0.24*).

Overall taste showed a weak positive correlation only with glucose (0.24*).

## 3. Discussion

Chlorophyll and carotenoid assays showed a significant effect of the cultivar. Results of chlorophyll a, chlorophyll b, and total chlorophyll content are consistent with the literature data [31]. Among the red cultivars, ‘Murai’ and ‘Carmesi’ showed the highest content of chlorophyll b and carotenoids, respectively, whereas the green cultivar ‘Kiribati’ exhibited the highest chlorophyll a content. Comparisons between green and red counterparts of the same lettuce type revealed significantly higher chlorophyll b (Oak type), total chlorophyll (a + b), and carotenoid content (Lollo type) in the red cultivars. Similarly, red lettuce showed a higher chlorophyll b content compared to green lettuce [32].

Biofertiliser treatments did not significantly affect chlorophyll b or total chlorophyll (a + b) content, while EM Aktiv and Vital Tricho treatments specifically increased chlorophyll a content by 28.6% and 23.8%, respectively.

In our study, lettuce plants were cultivated in soil characterised by low total nitrogen, high phosphorus, medium potassium, and medium organic matter content, under average suboptimal temperatures with a short-day photoperiod. EM Aktiv consists of beneficial bacteria from genera such as *Lactobacillus*, *Rhodopseudomonas*, *Bacillus*, and *Azotobacter*, as well as yeast, which promote plant growth by directly fixing atmospheric nitrogen or enhancing nitrogen availability via organic nitrogen mineralisation. These microorganisms improve soil fertility and plant growth by fixing nitrogen, decomposing organic matter, solubilising phosphate, and fostering beneficial microbial communities. Additionally, they protect plants from pathogens and increase stress tolerance [33]. Inoculation with *Bacillus* sp. VWC18 significantly enhanced lettuce and basil growth, doubling to tripling root weight, increasing chlorophyll content, and improving mineral uptake in a dose-dependent manner [34]. The nitrogen-fixing bacteria in EM Aktiv likely compensate for soil nitrogen deficiency by converting atmospheric nitrogen into plant-available forms or by enhancing nitrogen mineralisation, thereby supporting chlorophyll synthesis, photosynthesis, and overall metabolic functions. When pH shifts to acidified (5.5–6.5) or alkalised (7.5–8.5) ranges, pH becomes the primary controlling factor influencing bacterial community structure and functional dynamics [35] in addition to nutrient availability in the soil; this is especially important in our experiment, where the soil pH was measured at 8.1.

Vital Tricho, a biofertiliser containing *T. viride* and *T. asperellum*, also plays a critical role in enhancing nutrient availability. The literature supports that *Trichoderma* spp. positively regulates plant physiological processes such as photosynthesis, stomatal conductance, nutrient uptake, and water use efficiency, while promoting root growth and mineral nutrition [36,37]. Specifically, *T. viride* effectively solubilises insoluble inorganic and organic phosphorus, promoting growth in *Melilotus officinalis* [38] and increasing melon resistance to damping-off disease [39]. The consortium of *T. asperellum* T36b and *T. harzianum* Td50b similarly improved plant metabolism and yield in *Passiflora caerulea* [40].

The literature reports on chlorophyll and carotenoid content following biofertiliser application are inconsistent. Studies on lettuce and spinach treated with *Trichoderma asperellum* strains and *Trichoderma harzianum* in a floating root system also showed no significant changes in chlorophylls and carotenoids [41,42]. Similarly to our study, the application of microalgal-based biofertiliser treatments showed no significant increase in chlorophyll b content [43]. According to these authors, chlorophyll b may be less sensitive to variations in nutrient availability influenced by biofertilisers. This is further supported by the fact that chlorophyll b functions primarily as an accessory pigment, capturing light energy and transferring it to chlorophyll a, which is the main pigment involved in photosynthesis. On the contrary, the application of nitrogen combined with *Azotobacter* significantly enhanced chlorophyll content in lettuce [44]. The variation in results observed with different biofertilisers in our study can be explained by the existence of a balance in chlorophyll content rather than a simple increase or decrease, which could be better clarified by measuring photosynthetic efficiency and gas exchange.

Cultivar significantly affects TAC and TPC, with red lettuce cultivars consistently exhibiting higher levels than green ones. The red cultivar ‘Carmesi’ exhibits the highest TAC and TPC, consistent with our previous findings that identified this cultivar as having the highest TPC in soil rich in organic matter [6]. This pattern is supported by studies indicating that red cultivars accumulate more phenolics compared to green cultivars and the influence of genotype on the TPC [45,46].

Application of biofertilisers led to an increased level of TPC, while there was no significant effect on TAC. Consistent with our findings, the application of biostimulants such as Isabion (biostimulant rich in free amino acids and peptides) and Bactiva (containing different beneficial bacteria (*Bacillus*, *Pseudomonas*) and fungi (*Trichoderma* and *Gliocladium*, vitamins, plant protein amino acids, soluble extracts of *Yucca schidigera* and *Ascophyllum nodosum*)) showed no effect on TAC in lettuce [47]. Similarly, in lettuce, antioxidant capacity tended to decrease with arbuscular mycorrhizal fungi (AMF) treatment, possibly due to less stressful growth conditions, although the exact cause remains unclear [48]. In contrast, the use of fertilisers like EM Aktiv and combined fertilisers in our study resulted in increased TPC by 18.6% and 19.6%, respectively. Additionally, extracts from *Moringa oleifera* leaves significantly increased TPC in lettuce leaves compared to the untreated control [49]. A fulvic acid treatment (FA 40) also produced the highest levels of total phenols [4]. Furthermore, amino acid-based biostimulant Perfectose enhanced TPC [50]. Contrary to our results for *Trichoderma*, the application of AMF in lettuce has been reported to increase TPC [51].

Correlation coefficients between TAC and TPC showed a weak positive correlation (r = 0.40**) between these two parameters. These results suggest that phenolics contribute only partially to antioxidant activity, while other compounds also play a significant role. Positive correlations between TAC and TPC were previously reported in lettuce leaves treated with humic acid and glutamate [52]. Interestingly, sucrose exhibited a higher correlation coefficient with TAC, as sugar-derived compounds may contribute significantly to antioxidant activity via phenolic biosynthesis under low nitrogen conditions [53].

Carbohydrates, synthesised through photosynthesis, serve as energy sources for vegetative growth and development as well as precursors in the biosynthesis of various compounds, including polysaccharides, lipids, and proteins [54]. Previous studies have identified glucose, fructose, and sucrose as the three main sugars present in lettuce [55,56]. Fructose was identified as the predominant sugar in our analysis, with myo-inositol found alongside other sugars. Myo-inositol, a naturally occurring compound in many plants, has been extensively researched for its potential benefits in animal and human health, showing promise in conditions such as diabetes, insulin resistance, anxiety disorders, and depression [57]. Fructose is also the dominant sugar in various *Brassica* varieties [10]. On the other hand, sucrose acts as the primary transported sugar within plants, moving to different tissues where it can be either stored or utilised. Invertases cause its degradation, producing glucose and fructose, both of which are activated by hexokinases before being used. Alternatively, sucrose can be split by sucrose synthase, where only fructose needs this activation, and the other product, UDP-glucose, can be used more easily. Our study revealed two unidentified sugars similar to arabinose and maltose, but HPLC standards for these compounds did not show a positive match in peaks. The literature data on lettuce exposed to certain stressors, such as drought or contaminants, suggest the presence of maltose and arabinose under those scenarios [58,59].

The six tested cultivars significantly influenced the sugar profile, including TSS and TSI. The red cultivar ‘Carmesi’ exhibited the highest levels of glucose, fructose, sucrose, TSS, and TSI. Sugar accumulation and relative concentrations depend on various factors, including genotype and environmental conditions, indicating fructose as the predominant sugar in cabbage cultivated during the autumn [60]. Our results showed that in red Lollo-type, sucrose and TSS concentrations were higher compared to their green counterparts, which contrasts with the findings for TSS reported by Vargas-Arcila et al. [61].

The application of biofertilisers in our experiment showed statistically significant effects, except for their influence on sucrose content. Their impact on individual sugar levels was inconsistent. Notably, the combined fertiliser treatment led to decreased levels of glucose (by 24%), fructose (by 24.6%), and myo-inositol (by 17.1%), while the application of Vital Tricho specifically reduced fructose content by 17.4%. This may be because biofertiliser-treated plants utilise sugars like glucose and fructose more efficiently as energy sources and metabolic precursors for growth, resulting in lower residual sugar levels compared to the untreated control. Metabolically, decreases in glucose and fructose under biofertiliser treatments, especially Vital Tricho and combined treatment, indicate rapid sugar consumption to fuel respiration, specialised metabolite biosynthesis (e.g., phenolics and lactones), and stress response pathways under suboptimal temperature and/or nitrogen limitation. Contrastingly, untreated controls accumulate sugars due to lower metabolic utilisation. Elevated myo-inositol levels with EM Aktiv reflect its function as an osmoprotectant and antioxidant, maintaining osmotic balance and scavenging reactive oxygen species, which has been shown to improve photosynthesis, antioxidant enzyme activity, and membrane stability in quinoa under stress conditions [62]. Low nitrogen conditions in lettuce showed a reduced citrate cycle and higher glucose and sucrose levels, which led to an excess of carbon available for biosynthesis of phenolic compounds, as reported by Zhou et al. [63]. Sucrose and glucose levels increased in lettuce plants because they were not utilised as carbon skeleton sources for nitrogen assimilation [55]. On the other hand, EM Aktiv specifically increased levels of myo-inositol, and two unidentified sugars compared to the untreated control plants. Similar to our findings, the application of wood distillates, alone and in combination with soy lecithin and 5% flavonoid-rich wood glycolic extract, had no significant effect on sucrose content in lettuce [64].

Soluble solids are important quality attributes due to their influence on taste [61]. Carbohydrate content and composition play major roles in determining both flavour and overall vegetable quality [65]. Additionally, glucose is closely related to the perception of sweetness [18]. Our TSS results align with those observed in lettuce treated with seaweed-based biostimulants [29]. We found that combined fertiliser application led to higher TSS levels compared to the untreated control. In contrast, tendril pea showed a 13.15% decrease in TSS following combined application of *Bacillus thuringiensis* and *Trichoderma asperellum*, which may slightly affect sensory attributes but could indicate a metabolic shift toward other beneficial phytochemicals [66]. Additionally, microbial fertiliser application in lettuce led to decreased TSS compared to the untreated control plants [67]. Application of *Ecklonia maxima* extracts in hydroponic leaf lettuce cultivation showed no significant effect on soluble solids, although some concentrations were higher than the control [68]. This aligns with our observations for individual applications of EM Aktiv and Vital Tricho, where values were higher than the control but not statistically significant.

Soluble sugars in lettuce mainly consist of fructose, glucose, and sucrose, with the total sweetness index (TSI) determined by the sweetness coefficient and concentration of each sugar [69]. Our TSI results were lower than those reported in lettuce exposed to 16 h of monochromatic red light during the 16 h photoperiod [65]. This difference is likely due to the shorter photoperiod in our study (11–9 h), as environmental factors like photoperiod length can affect TSI. The short-day photoperiod during the autumn experiment likely limited the duration of photosynthetic activity, thereby reducing sugar synthesis in lettuce, consistent with findings that light duration and quality directly regulate carbohydrate accumulation and enzyme activities involved in sucrose metabolism [65]. Total soluble solids increased with combined treatment despite reductions in glucose and fructose, suggesting accumulation of other compounds, such as organic acids and phenolics, which contribute less to sweetness, thereby explaining the decline in total sweetness index.

Correlation coefficients revealed positive relationships among the three dominant sugars. Similarly, positive correlations between glucose and fructose were observed in various lettuce cultivars [70] and cabbage cultivars [60]. Our study also found positive correlations between sucrose and both glucose and fructose, suggesting that the synthesis of one sugar enhances sucrose content. In contrast, results in cabbage showed either negative or no significant correlations between glucose and fructose with sucrose, reflecting differences in how sucrose is converted into glucose and fructose [60].

Organic acids are key acidic components in fruits and vegetables that directly influence their taste, flavour, and nutritional quality. They play essential roles in photosynthesis, respiration, metabolism, and the synthesis of phenols, amino acids, esters, and aromatic compounds. Malic and citric acids are usually the most abundant organic acids in many fruits and vegetables. The anions of citric and malic acids are intermediates of the Krebs cycle, while tartaric acid is synthesised from ascorbic acid [71]. Cultivar significantly affected the organic acid profile, with Oak-type cultivars generally exhibiting the highest levels of these predominant acids. Organic acid concentration in lettuce depends on the cultivar, with the main acids identified as malic, tartaric, citric, and oxalic acid [72], which aligns with our sample profile.

The application of biofertiliser did not affect the content of most organic acids; however, treatment with EM Aktiv significantly increased the levels of propionic acid by 29.7% and two unidentified organic acids by 25% and 12.5%. Biofertilisers used in our study, including *Trichoderma*, are primarily known to enhance nutrient availability and absorption by improving soil nutrient solubility and promoting root growth, rather than directly influencing the metabolic pathways responsible for the synthesis of organic acids within the plant. Organic acid levels remained stable across treatments, signifying maintenance of primary metabolism and adaptive homeostasis under suboptimal conditions. EM Aktiv notably elevated propionic acid, likely due to enhanced microbial activity on leaf surfaces, especially in the moderately alkaline soil (pH 8.1), where root uptake of this acid is limited. Literature reports show inconsistent findings regarding organic acid content under different biostimulant applications. For instance, plant-based biostimulants have been found to increase malate and citrate levels but not significantly affect tartaric acid [73]. Similarly, these authors observed higher biosynthesis of citric and tartaric acids in the green cultivar compared to the red cultivar, potentially due to the regulatory role of organic acids as anti-anions for nitrates. This pattern was also reflected in our results for tartaric acid between green and red Salanova cultivars. In aquaponic lettuce cultivation, treatments with *Azotobacter chroococcum* led to increased levels of organic acids such as citric, malic, succinic, and isocitric acids—intermediates of the TCA cycle—indicating an activation of energy production pathways, whereas treatments without *Azotobacter* resulted in either no significant change or a decrease in organic acids compared to hydroponics [74]. Organic acids serve as important indicators of plant tolerance to stress, including nutrient deficiencies. In spinach under nitrate stress, fulvic acid treatment increased the contents of tartaric and malic acids, which supply essential substrates for the TCA cycle and amino acid biosynthesis, thereby supporting normal energy production and amino acid synthesis [16].

All major organic acids showed positive correlations among themselves. These results indicate that an increase in the level of one organic acid may enhance the levels of others. However, the literature data present slightly different patterns; for example, there is a positive correlation specifically between tartaric and malic acids [16].

Polyphenols, carbon-based specialised metabolites, play vital roles in plant–environment interactions. Their biosynthesis mainly occurs through the shikimate and phenylpropanoid pathways, with phenylalanine and tyrosine serving as key precursors. Ubiquitous in plants, phenolic compounds are integral to the human diet and attract considerable interest for their antioxidant properties and potential health benefits [75]. However, their health-promoting effects depend on the amount consumed as well as on their bioavailability. Additionally, these compounds influence food quality by affecting sensory attributes such as bitterness, astringency, and herbaceous flavours, thereby impacting consumer acceptance [76].

The lettuce phenolic acids profile reveals the presence of chicoric acid, caffeoylquinic acid glucoside, gallic acid, and chlorogenic acid as major phenolic acids. The red cultivars ‘Murai’ and ‘Carmesi’ exhibited the highest levels of most phenolic acids. Comparing the contribution of each compound and considering leaf colour, red cultivars generally exhibited higher levels of most phenolic acids, except caffeic acid. Caffeic acid derivatives were the main phenolics in green varieties, while flavonols were detected in higher quantities in red varieties [46]. Chicoric acid is a characteristic phenolic acid commonly found in the Asteraceae family, while chlorogenic acid is widely distributed across various plant species [77]. Previous reports showed that chicoric acid was the predominant phenolic acid found in lettuce samples [8,78]. Chlorogenic acids are formed through the esterification of trans-cinnamic acids and quinic acid. Their biosynthesis is influenced by environmental conditions and agricultural practices. Chlorogenic acids are also recognised for their protective effects against oxidative stress-related diseases in humans, including cancer, diabetes, inflammation, and cardiovascular diseases [79]. Coumaroylquinic acid was the second most common phenolic compound in lamb’s lettuce [80], which was also detected in our samples.

The major flavonoids identified in our samples were different quercetin derivatives, including quercetin-3-O-(6″-O-malonyl)-glucoside, quercetin-3-glucoside, quercetin-3′-O-glucuronide, and quercetin-3-O-(6″-malonyl-glucoside)-7-O-glucoside, as well as kaempferol and its derivative. Similar to our results, different quercetin derivatives were found to be major in lettuce samples of the red Lollo cultivar with the highest content of polyphenols [78]. Quercetin is well-known for its diverse bioactive properties, such as anti-cancer, anti-bacterial, anti-viral, anti-inflammatory, anti-diabetic, and cardiovascular disease-prevention effects [81]. Kaempferol exhibits therapeutic potential in managing neurodegenerative diseases, cardiovascular disorders, and metabolic syndromes [82].

Cultivar showed significant effects on all phenolic acids and flavonoids, while biofertiliser had no effect except on gallic acid and kaempferol. Cultivar and lettuce type are found to influence phytochemical and polyphenol composition [8]. Specifically, lettuce plants with intense red leaf colouration, undulating leaves, and densely incised leaf margins tended to accumulate the highest concentrations of flavonoids and hydroxycinnamic acids [83]. This finding aligns with the HPLC results from our trial on red cultivars ‘Carmesi’ and ‘Murai’, which exhibited similar leaf morphology and phytochemical profiles.

Apart from the genotype, polyphenol content in lettuce was influenced by different growing treatments varying in the availability of the nutrients, where a quarter-strength reduced-nutrient solution increased the accumulation of polyphenols compared to the full-strength solution [84]. Abiotic stress conditions activate the phenylpropanoid biosynthetic pathway, leading to an increase in various phenolic compounds that enhance plant performance under stress. The effect of nitrogen limitation on phenolic accumulation is still not well understood, with several mechanisms proposed, such as the carbon/nitrogen (C/N) balance theory, deamination theory, and photoprotection theory. These theories suggest that nitrogen limitation causes the plant to accumulate carbon-based assimilates by activating specialised metabolite biosynthesis [85]. Our results for gallic acid showed that treatments did not differ from the control plants, yet there was a difference between biofertiliser treatments, where EM Aktiv and combined fertiliser led to higher levels compared to the application of Vital Tricho. Our study was conducted on soil characterised by low total nitrogen levels and medium organic matter content. A decrease in soil chemical compound availability can shift plant metabolism toward increased synthesis of specialised metabolites, such as phenolic acids and flavonoids, often at the expense of primary metabolites [86]. Cultivating lettuce in a conventional system led to higher phenolic content and could be associated with the presence of more stressful conditions in terms of plant and/or soil mineral deficits [87]. When stress is low, the plant allocates metabolic resources toward polyphenols that support growth and development rather than plant defence mechanisms [52]. However, our results do not fully align with the results observed in the organic soil cultivation. Interestingly, only kaempferol showed a significant response to biofertiliser application in the range 12.5–25%, while other phenolic acids and flavonoids were not significantly affected. Kaempferol levels increased across all biofertiliser treatments and suboptimal temperature conditions, reflecting its role as a potent antioxidant and signalling molecule in plant stress tolerance. This flavonol is upregulated in oxidative stress and pathogen defence and has been linked to recruitment of beneficial Bacillaceae to roots, facilitating nitrogen acquisition and enhanced nutrient uptake [88]. Additionally, *T. asperellum* induced resistance of tomato plants against the root-knot nematodes by systemically promoting specialised metabolites such as kaempferol in distant roots [89].

Although many compound levels were higher than in the control (untreated) plants, these differences did not reach statistical significance. Our previous research on a fertile soil also showed that biofertilisers did not significantly affect chicoric acid and quercetin-3-malonylglucoside-7-glucoside [6]. Application of AMF increased the levels of chlorogenic, caffeic, and gallic acids, while quercetin content remained unaffected [51]. Differences were found between untreated plants and treatments with the *Candida guillermondii* strain, which showed a tendency to increase the levels of the majority of phenolics, especially chicoric acid and quercetin derivatives [90]. Results related to different sulphur nutrition showed that chicoric acid, caffeic acid derivatives, and various quercetin derivatives exhibited no significant changes [91]. Many authors report that plant-derived biostimulants activate specialised metabolism by increasing the expression of genes encoding phenylalanine (tyrosine) ammonia-lyase enzymes. Phenylalanine is converted by the enzyme phenylalanine ammonia-lyase into trans-cinnamic acid, which is then hydroxylated by cinnamate 4-hydroxylase to form *p*-coumaric acid, marking the starting point for the biosynthesis of flavonoids [73].

Sesquiterpene lactones are a key group of specialised metabolites predominantly found in the Asteraceae family, where they play vital roles in plant defence and contribute significantly to the characteristic bitterness of lettuce. These compounds interact with human bitter taste receptors (*hTAS2R46*) [92] and can account for over 2% of the plant’s dry leaf weight [93]. Their concentration and composition vary by lettuce type and genetic background [18], as demonstrated in different Little Gem cultivars exhibiting distinct lactone levels [94].

In our study, nine sesquiterpene lactones were identified across six cultivars, though 11β,13-dihydro-8-deoxylactucin was absent in most of the samples. Lactucin, lactucopicrin-15-oxalate, and 11β,13-dihydrolactucin were most abundant, while lactucopicrin ranked fifth, consistent with previous reports identifying lactucopicrin, lactucin, 11β,13-dihydrolactucopicrin, and 8-deoxylactucin as major lettuce lactones [91,95]. Lactucopicrin, with the lowest bitter taste threshold, contributes over 72% of the perceived bitterness activity value [96].

Significant cultivar differences emerged: the red cultivar ‘Carmesi’ exhibited the highest peak areas for most lactones, except lactucin, 11β,13-dihydrolactucin-15-glucoside, and 11β,13-dihydrolactucin, which were enriched in red ‘Gaugin’ and green ‘Kiribati’, respectively. Our earlier work with these cultivars grown in soil characterised by high organic matter content and medium total nitrogen also identified lactucopicrin as the dominant lactone but found lactucin and 11β,13-dihydrolactucin present inconsistently [6], emphasising soil’s influence on lactone profiles through plant metabolism and nutrient availability.

Application of studied biofertilisers significantly affected lactone peak areas. EM Aktiv notably increased 8-deoxylactucin by 185.7%, 11β,13-dihydrolactucin by 32.7%, lactucopicrin by 25%, lactucopicrin-15-oxalate by 47.5%, and total lactones by 14.7%. Vital Tricho raised lactucin peak areas by 26.4%, while combined fertilisation enhanced several lactones, including 8-deoxylactucin (128.6%), lactucin (32.1%), lactucopicrin-15-oxalate (25%), 11β,13-dihydrolactucopicrin-15-oxalate (28%), and total lactones (12.5%). These findings suggest that biofertilisers can modulate bitter compound levels, potentially impacting both taste and nutraceutical value.

Our previous research in soil characterised by distinct chemical and physical properties corroborated these effects, showing that combined fertilisation elevated lactucopicrin and total lactones, while all biofertilisers increased 11β,13-dihydrolactucopicrin [6]. However, broader knowledge on fertilisation impacts remains limited and sometimes inconsistent. No significant changes have been observed with varying sulphur nutrition [91]. Additionally, lactucopicrin levels vary by production system—soil, substrate, or hydroponics—and nitrogen form, with the highest levels in soil-grown lettuce under 25% NH_4_^+^ and 75% NO_3_^−^ [97]. Unlike phenolics and flavonoids, whose levels might be stable or less responsive to these microbial stimuli in certain soil conditions, sesquiterpene lactones act as phytoalexins or antimicrobial agents, and biofertilisers may selectively enhance sesquiterpene lactone production to strengthen plant defence without significantly altering phenolic acids or flavonoid levels.

Lactones, known as bioactive specialised metabolites involved in plant defence and microbial signalling, broadly increased in treated plants. Their antimicrobial action and role as signalling molecules mediate beneficial plant-microbe interactions stimulated by biofertilisers and environmental stress. EM Aktiv might promote lactone production through enhanced nutrient availability and activation of metabolic pathways like the mevalonate pathway, while *Trichoderma* spp. activate defence-related signalling and gene expression [98], leading to increased production of defence compounds such as lactucin [13]. These mechanisms collectively illustrate how beneficial microbes shape plant metabolism to enhance growth, defence, and stress responses. Future field studies and simulations mimicking natural conditions are required to further understand the regulation of sesquiterpene lactones biosynthesis under environmental variables [99].

Overall taste remains one of the most crucial factors influencing consumer choice, along with size, texture, and rosette diameter. While individual sugars contribute to the sweetness of lettuce, sesquiterpene lactones play a key role in its bitterness, which has a particularly strong effect on consumer preference [100].

Based on sensory panel evaluations, all lettuce cultivars scored between 2.54 and 3.08 on a bitterness scale ranging from bitter to neutral. Both the cultivar type and biofertiliser application significantly influenced overall taste. The green cultivar ‘Aquino’ was rated as having the most pleasant overall taste compared to the green cultivar ‘Aleppo’. ‘Aquino’ exhibited the lowest levels of several key compounds, including the most abundant lactones (lactucin, lactucopicrin, total lactones, lactucopicrin-15-oxalate), fructose, citric acid, quercetin derivatives, kaempferol glucoside, chlorogenic acid, and caffeic acid, which may explain these results.

However, the literature suggests that bitterness perception and consumer liking are largely determined by the balance between bitter sesquiterpene lactones and sweet sugars, rather than by the concentration of individual compounds [18]. The presence of sugars can mask and counterbalance bitterness [101]. However, concerns about sugar content, especially in ultra-processed foods, are increasing [102]. Despite this, sugar remains an important breeding trait due to its strong association with consumer acceptance.

Overall taste showed only one positive correlation with the analysed compounds—a weak positive correlation with glucose. This aligns with the observation that glucose was correlated with sweetness perception, despite fructose being the sweetest sugar present [18]. Lactucin exhibited weak positive correlations with chlorophyll b and total chlorophyll, and a weak negative correlation with fructose, suggesting that as lactucin increases, fructose decreases. Lactucopicrin showed no significant correlations with the predominant sugars or chlorophyll, while lactucopicrin-15-oxalate displayed weak positive correlations with glucose, fructose, and sucrose, indicating that sugar content increases alongside it. These results partially support findings of Seo et al. [96], which reported negative and positive correlations of chlorophyll and sugar content with lactucin, respectively, but no significant relationships with lactucopicrin or 8-deoxylactucin. This implies that chlorophyll and sugar levels may influence lactucin concentration and, consequently, bitterness in lettuce. Lactucopicrin and kaempferol malonyl glucoside were consistently linked to bitterness, astringency, and herbaceous flavour perceptions, with overall acceptance negatively impacted mainly by bitterness [76]. Our results revealed a moderate positive correlation between lactucopicrin and kaempferol malonyl glucoside levels, which may jointly influence taste.

The findings of this study provide specific insights into the effects of cultivar and biofertiliser treatments, partially confirming the proposed hypotheses. The first hypothesis was proven, as the cultivar significantly influenced all tested parameters, while biofertilisers affected specific metabolite groups, such as chlorophyll a, total carotenoids, sugars, TSI, TSS, certain organic acids, TPC, lactones, and overall taste. In support of the second hypothesis, which was partially confirmed, biofertiliser application elevated levels of chlorophyll a, myo-inositol, two unidentified sugars, two unidentified organic acids, and propionic acid, TSS, TPC, kaempferol, and key lactones. Regarding the third hypothesis, a synergistic effect of both fertilisers was evident in a few metabolites, including lactucin, 11β,13-dihydrolactucopicrin-15-oxalate, and TSS, while showing a rather additive effect on TPC. Furthermore, the fourth hypothesis was confirmed since the combined fertiliser produced the most favourable taste, achieving the highest score and a significant increase compared to Vital Tricho, although it did not differ significantly from the control.

Considering the previous discussion, our study has several potential limitations. It examined one season, leaving the long-term effects of biofertilisers on primary and specialised metabolites unexplored. The study primarily examined the effects of two main factors, with limited exploration of their interactions. Although biofertiliser did not affect certain metabolites, significant cultivar × fertiliser interactions were detected for nearly all tested parameters, except total carotenoids, tartaric acid, and caffeic acid and its derivatives. These interactions warrant more comprehensive investigation.

## 4. Materials and Methods

### 4.1. Plant Material and Biofertilisers

Six lettuce cultivars from Rijk Zwaan company (De Lier, the Netherlands) were included in the experiment, representing three types: Oak leaf (green ‘Kiribati’ and red ‘Murai’, *L. sativa* var. *crispa*), Lollo (green ‘Aleppo’ and red ‘Carmesi’, *L. sativa* var. *crispa*), and multi-leaf butterhead Salanova^®^ (green ‘Aquino’ and red ‘Gaugin’, *L. sativa* var. *capitata*). Seedlings were cultivated in mechanically formed 4 cm peat cubes made from Potgrond H substrate (Klasmann-Deilmann GmbH, Geeste, Germany) and grown under glasshouse conditions in Irig, Serbia (45°04′12″ N, 19°51′25″ E; 159 m above sea level). Following a 20-day seedling production period, plants with four fully expanded leaves were transferred to the greenhouse for further cultivation.

Two different biofertilisers were applied in this study. EM Aktiv (Candor, EM tehnologija d.o.o., Valpovo, Croatia) is a liquid medium containing plant extracts obtained through the microbiological fermentation of sugar cane molasses and organic matter. Vital Tricho (Candor, EM tehnologija d.o.o., Valpovo, Croatia) is a powdered formulation composed of two species, *Trichoderma viride* and *Trichoderma asperellum* (5 × 10^9^ CFU mL^−1^). The third treatment consisted of simply physically mixing EM Aktiv and Vital Tricho.

### 4.2. Experimental Design and Environmental Conditions

An autumn experiment was conducted from October to December in a greenhouse area of 144 m^2^, without additional heating and lighting, located approximately 12 km from the city centre of Belgrade, Serbia (44°47′39″ N, 20°20′12″ E; 73 m above sea level). Prior to the experiment, the soil, classified as a Hortic Anthrosol (Terric, Transportic) [103], formed during the excavation of an ameliorative channel, had been intensively used for vegetable production without biofertiliser application. Mechanical and chemical soil analysis identified the soil as sandy clay loam with a moderately alkaline pH (H_2_O = 8.1). It contained low total nitrogen (0.14%), high readily available phosphorus (33.92 mg 10^−2^ g^−1^), medium readily available potassium (18.69 mg 10^−2^ g^−1^), and moderate soil organic matter content (2.83%).

**Figure 1:**
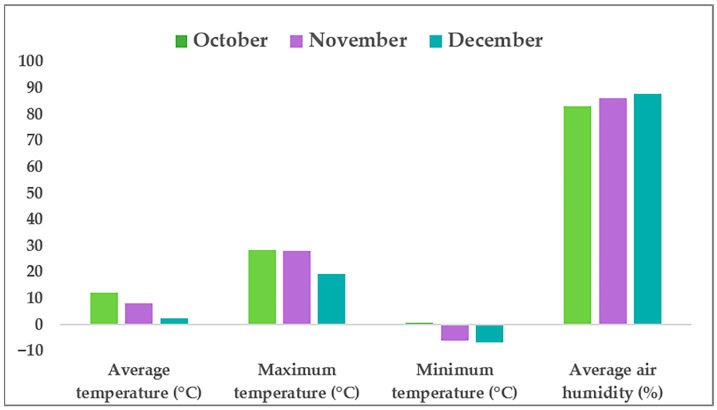
Climate conditions during lettuce growing period.

The trial was organised in a complete block design with four treatments as follows: (a) control (without applying any fertiliser), (b) the fertiliser EM Aktiv, (c) the fertiliser Vital Tricho, and (d) combined fertilisers EM Aktiv and Vital Tricho, in three replicates. Each plot measured 0.75 m × 1 m and contained 12 plants cultivated at a density of 25 cm × 25 cm. The spacing between repetitions within each treatment was 50 cm, and the distance between treatments was 100 cm. Biofertilisers were applied directly to the soil after preparation with a tractor and tiller (75 mL of EM Aktiv, 10.5 g of Vital Tricho, and a combination of 75 mL + 10.5 g of EM Aktiv and Vital Tricho, each dissolved in 5 L of water) and during the vegetation period foliarly four times using a battery sprayer (15 mL of EM Aktiv, 6 g of Vital Tricho, and 15 mL + 6 g of EM Aktiv and Vital Tricho were dissolved in 3 L of water). After soil preparation and application of the fertilisers to the soil, black mulch film was set up. Plants were cultivated using regular agricultural measures during the growing cycle. Climate data, including air temperature and humidity, were continuously monitored over 24 h using an RC-4HC Data Logger (Elitech Technology Inc., San Jose, CA, USA) (Figure 1) during a short-day photoperiod that varied from 11 to 9 h between the beginning and the end of the experiment.

### 4.3. Data Collection and Sample Preparation for Spectrophotometric Assays

Harvest timing was determined by physiological maturity alongside marketable rosette appearance (compactness, size). Fresh lettuce leaves were stored in plastic bags at −20 °C in a freezer until further analysis. Frozen whole lettuce rosettes, excluding the stems, were ground in liquid nitrogen. Approximately 0.5 g of the homogenised leaf tissue was extracted in 1.5 mL of 80% methanol, followed by centrifugation at 13,000× *g* for 10 min to obtain a supernatant for TAC and TPC assays. To obtain supernatant for chlorophyll and carotenoid analysis, 0.5 g of frozen tissue was homogenised and extracted with 1 mL of 80% acetone, then centrifuged at 13,000× *g* for 10 min at 4 °C.

#### Spectrophotometric Assays—Total Antioxidant Capacity, Total Phenolic Content, Chlorophyll and Carotenoids Content

TAC was measured using the ABTS assay [104]. The reaction mixture included 2 mM ABTS, 15 μM H_2_O_2_, and 0.25 μM horseradish peroxidase (HRP) in 50 mM phosphate buffer at pH 7.5. The reaction was recorded at 730 nm (Multiskan Spectrum, Thermo Electron Corporation, Vantaa, Finland) at 25 °C until the absorbance stabilised, as a consequence of ABTS^+^ radical formation. The addition of varying concentrations of ascorbic acid (0.1–0.8 mM) and sample extracts to the reaction mixture caused a decrease in absorbance due to the scavenging of ABTS^+^ radicals. This change in absorbance was quantified using a linear regression equation derived from the standard curve, and the results were expressed as milligrams of ascorbic acid equivalents per gram of fresh weight (mg AA eq g^−1^ FW).

TPC was assessed by the Folin–Ciocalteu spectrophotometric method, with gallic acid (GA) used as the standard to create the calibration curve [105]. Standards and samples were combined with 0.25 N Folin–Ciocalteu reagent and incubated for three min at room temperature. Next, 0.2 M sodium carbonate solution was added, followed by a 60 min incubation at room temperature. Absorbance was measured at 724 nm using a Multiskan Spectrum spectrophotometer (Thermo Electron Corporation, Vantaa, Finland), and results are expressed as micrograms of gallic acid equivalents per gram of fresh weight (μg GA eq g^−1^ FW).

Chlorophyll and carotenoid contents were spectrophotometrically measured from acetone extracts following a previously reported method [106]. The absorbance of the extraction was recorded at 470 nm, 647 nm, and 663 nm using a Multiskan Spectrum spectrophotometer (Thermo Electron Corporation, Vantaa, Finland). The concentrations of chlorophyll and carotenoids were calculated using the following equations:

Chl_a_ = 12.25 × A663 − 2.79 × A647 (μg mL^−1^) (1)

Chl_b_ = 21.5 × A647 − 5.1 × A663 (μg mL^−1^) (2)

Chl_(a + b)_ = 7.15 × A663 + 18.71 × A647 (μg mL^−1^) (3)

Chl_x+c_ = (1000 × A470 − 1.82 × Chl_a_ − 85.02 × Chl_b_)/198 (μg mL^−1^) (4)

Results for chlorophyll a, chlorophyll b, chlorophyll (a + b), and total carotenoids are expressed as micrograms per gram of fresh weight (μg g^−1^ FW).

### 4.4. Sample Preparation for the HPLC Analysis

Similarly to the preparation for spectrophotometric assays, frozen samples were first ground under liquid nitrogen. The resulting powder was then homogenised and extracted in 80% methanol at a ratio of 1:3 (*w*/*v*). After homogenisation, centrifugation was performed at 13,000× *g* for 10 min at 4 °C, and the supernatant was used for further HPLC analysis of sugars and organic acids. Before HPLC analysis of phenolics and sesquiterpene lactones, the samples underwent lyophilisation for 48 h. Once freeze-dried, samples were sealed in plastic bags and stored at −20 °C until further analysis.

For individual phenolic compounds, freeze-dried samples underwent extended maceration using an ultrasonic processor (USCG-300, Infitek Co., Ltd., Jinan, China). The extraction was performed in 80% methanol at a 1:3 (*w*/*v*) ratio for 5 min. After that, samples were shaken at 1000 rpm (Heidolph Instruments GmbH & Co. KG, Schwabach, Germany) for 17 h at 25 °C. Then the mixture was centrifuged at 11,000× *g* (Sigma Centrifuge, Sigma Laboratory Centrifuges, Osterode, Germany) for 30 min at 25 °C, and the supernatant was used for further HPLC analysis.

For sesquiterpene lactone analysis, 100 mg of freeze-dried powdered samples were extracted with 1 mL of water/methanol mixture (80:20, *v*/*v*), briefly vortexed, and then shaken at 1400 rpm (Thermomixer C, Eppendorf, Montesson, France) for 15 min at 35 °C. After extraction, the mixture was centrifuged at 21,000× *g* (Sigma Centrifuge, Sigma Laboratory Centrifuges, Osterode, Germany) for 10 min at 4 °C. The resulting supernatants were finally filtered through a 0.45 μm PP Whatman UNIFILTER microplate (Cytiva, VWR, Rosny-sous-Bois, France) and transferred to vials for UPLC-UV analysis.

#### 4.4.1. HPLC Analysis of Sugars and Organic Acids

Sugar analysis was conducted utilising a CarboPac PA1 column, 250 × 4 mm, Dionex (Thermo Fisher Scientific, Waltham, MA, USA) paired with its corresponding guard column. The column was maintained at 30 °C and at a flow rate of 1.0 mL min^−1^ to achieve optimal separation. Detection was carried out using a Waters 2465 electrochemical detector, equipped with a 3 mm gold working electrode and a hydrogen reference electrode, providing highly sensitive and selective measurement of sugars under the applied chromatographic conditions. The mobile phase was 200 mM sodium hydroxide, prepared by diluting 10.5 mL of 50% (*w*/*w*) of low-carbonate sodium hydroxide solution (J.T. Baker, Deventer, The Netherlands) with vacuum-degassed deionised water to a final volume of 1 L. Detection was performed in pulse mode, integrating signals over 150 ms, using a waveform E1 = +0.15 V for 400 ms, E2 = +0.75 V for 200 ms, and E3 = −0.8 V for 200 ms. The filter timescale was set to 0.2 s, and the detection range was 1 μA. Data collection and analysis were conducted using Waters Empower 2 software (Waters, Milford, MA, USA). Peaks for myo-inositol, glucose, fructose, and sucrose were validated and quantified by the external standard method, expressed as milligrams per gram of fresh weight (mg g^−1^ FW). The two unidentified sugars, compound **1** and compound **2**, were expressed as milligrams of arabinose equivalents per gram of fresh weight and milligrams of maltose equivalents per gram of fresh weight, respectively. Equivalents were chosen based on the similar retention times of standards with unknown sugars.

Organic acids were separated on the same HPLC system (Waters, Milford, MA, USA) equipped with a 1525 binary pump, column thermostat, and 717+ autosampler, coupled to a Waters 2996 diode array detector set at 210 nm. Separation was performed on a Supelco C-610H column 300 × 7.8 mm (Sigma-Aldrich, Barcelona, Spain) with a corresponding guard column, using isocratic elution with 0.1% phosphoric acid as the mobile phase at a flow rate of 0.5 mL min^−1^ and a column temperature of 40 °C. Data collection and analysis were conducted with Waters Empower 2 software (Waters, Milford, MA, USA). Peaks for malic, citric, tartaric, and propionic acids were validated and quantified by the external standard method and expressed as milligrams per gram of fresh weight (mg g^−1^ FW), while shikimic and fumaric acids were expressed in micrograms per gram of fresh weight (μg g^−1^ FW). Similarly, the two unidentified organic acids, compound **3** and compound **4**, were expressed as milligrams of formic acid equivalents and oxalic acid equivalents per gram of fresh weight, respectively. Again, equivalents were chosen based on the similar retention times of standards corresponding to peaks of unknown compounds. All standards were obtained from Sigma-Aldrich (St. Louis, MO, USA).

##### Total Soluble Solids and Total Sweetness Index

After HPLC sugar analysis, the results for glucose, fructose, and sucrose were used to determine the total sweetness index (TSI). The TSI was calculated by the concentration and sweetness coefficient of each soluble sugar using the Equation [69]:

TSI = 1.50 × fructose + 0.76 × glucose + 1.00 × sucrose(5)

In this equation, each sugar is multiplied by a specific factor reflecting its perceived sweetness compared to sucrose.

Total soluble solids (TSS) of the juice were measured at room temperature using a digital hand refractometer (ORA20BA, Kern & Sohn GmbH, Balingen, Germany) following [107] and expressed as degrees Brix (°Brix).

#### 4.4.2. HPLC/DAD/MS Analysis of Phenolic Compounds

Samples were analysed using a Waters HPLC system equipped with a 1525 binary pump, thermostat, and 717+ autoinjector, coupled to a 2996 Diode Array Detector (DAD) and an EMD 1000 quadrupole detector with an electrospray ionisation (ESI) source (Waters, Milford, MA, USA). Phenolic compounds were separated on a Symmetry C-18 reversed-phase column (125 × 4 mm, 5 μm) with a corresponding pre-column. The mobile phases were 0.1% formic acid (A) and acetonitrile (B), delivered at 1 mL min^−1^ using the following gradient profile: starting at 10% B, increasing linearly to 50% B over 35 min, then returning to 10% B over 10 min, followed by 5 min of equilibration. A post-column flow splitter (5:1) (ASI, Richmond, CA, USA) was used to achieve a 0.2 mL min^−1^ optimal flow rate for MS detection. DAD detection was set at 330 nm for phenolic acids and 380 nm for flavonoids. Peak identification was confirmed by LC/MS analysis in negative ion scanning mode (100–900 *m*/*z*), with ESI source parameters as: capillary voltage 3.0 kV, cone voltage −35 V, extractor voltage 3.0 V, RF lens 0.2 V, source temperature 130 °C, desolvation temperature 400 °C, and nitrogen flow at 500 L h^−1^. Data were processed and peaks confirmed using Waters Empower 2 software (Waters, Milford, MA, USA). Gallic acid, chlorogenic acid, caffeic acid, and kaempferol were quantified using the external standard method (Sigma-Aldrich, St. Louis, MO, USA) and expressed as micrograms per gram of fresh weight (µg g^−1^ FW). Chicoric acid content was expressed as micrograms of caffeic acid equivalents per gram of fresh weight. Quercetin and kaempferol derivatives were quantified and expressed as micrograms of quercetin and kaempferol equivalents per gram of fresh weight, respectively. Other caffeic acid derivatives, caffeoylquinic acid, and *p*-coumaroylquinic acid were expressed as micrograms of caffeic acid equivalents and coumaric acid equivalents per gram of fresh weight.

#### 4.4.3. UPLC Analysis of Sesquiterpene Lactones

Sesquiterpene lactones in dried methanol extracts were quantified using an Ultimate 3000RS UPLC system (Thermo Fisher Scientific, Villebon-sur-Yvette, France) equipped with a quaternary pump, column oven, and UV-visible diode array detector. Separation was achieved on an Uptisphere Strategy PHC4 column (150 × 3 mm, 3 μm; Interchim, Montluçon, France). The mobile phases consisted of water (A) and acetonitrile (B), each acidified with 0.1% phosphoric acid, and were delivered at a flow rate of 0.7 mL min^−1^. The gradient elution was as follows: linear from 6% to 32% B for 32 min; linear from 32 to 85% B in 1 min; isocratic at 85% B for 2 min; linear from 85 to 6% B in 1 min; and isocratic at 6% B for 6 min. The oven temperature was set to 45 °C, and the injection volume was 5 μL. Ten sesquiterpene lactone compounds were profiled (lactucin, lactucin-15-oxalate, 8-deoxylactucin, 11β,13-dihydrolactucin, 11β,13-dihydrolactucin-15-glucoside, lactucopicrin, lactucopicrin-15-oxalate, 11β,13-dihydrolactucopicrin, 11β,13-dihydrolactucopicrin-15-oxalate, and 11β,13-dihydro-8-deoxylactucin), as determined by LCQTOF-HRMS analysis [108]. The total STL content was then expressed as the sum of the peak areas of these 10 compounds, as detected in the UV chromatogram at 254 nm.

### 4.5. Sensory Analysis

After the harvest, on the same day, a sensory panel was conducted with four panellists. Before the evaluation began, the panellists were given a brief overview of the testing procedure, including the context, rules, and method for assessing overall taste. The evaluators underwent training based on the general guidelines outlined in the ISO standard [109], which focus on selecting, training, and monitoring sensory assessors. As part of their preparation, panellists learned to identify and distinguish the primary taste qualities-bitterness, sweetness, saltiness, and sourness. They were provided with five different solutions, with water serving as the neutral control, and were instructed to rank the samples based on taste intensity, from the weakest to the strongest.

Lettuce samples were prepared in a way that any damaged or discoloured outer leaves were removed, and the samples were thoroughly washed and dried. Each panellist was seated separately and provided with an evaluation sheet and a pencil. To ensure objectivity, samples were coded with numbers and presented in plastic bags. Each treatment consisted of 12 samples for testing. Panellists were encouraged to describe their sensory impressions in detail to capture comprehensive feedback. Water and toasted bread were available for palate cleansing between samples. The overall taste of lettuce samples was assessed on a 5-point hedonic scale, ranging from 1 (very poor) to 5 (very good) [110].

### 4.6. Statistical Analysis

A two-way ANOVA, followed by Tukey’s post hoc test, was conducted to evaluate the effects of the main factors, cultivar and fertiliser, at a significance level α of 0.05. Pearson’s correlation was used to examine the relationships between the tested parameters. The results are presented in a heat map graph and a separate table highlighting statistical significance. The statistical analysis was performed using the software SPSS Statistics (Version 25.0; IBM Corp.: Armonk, NY, USA) and Microsoft Office Excel 2019 (Microsoft Corp., Redmond, WA, USA).

## 5. Conclusions

The red cultivars ‘Carmesi’ and ‘Murai’ demonstrated superior phytochemical attributes, with ‘Carmesi’ exhibiting the highest levels of predominant sugars, quercetin derivatives, lactucopicrin and its derivatives, TSI, TSS, TAC, TPC, and total carotenoids, while ‘Murai’ showed the greatest concentrations of predominant organic acids and phenolic acids. In contrast, the green cultivar ‘Aquino’ excels in sensory evaluation, offering the most favourable overall taste despite its lower metabolite concentrations.

While biofertiliser treatments did not affect major metabolites, their significant influence on sesquiterpene lactones, as well as their significant cultivar × fertiliser interactions across nearly all parameters, highlights the complex influence of biofertilisers on lettuce chemical composition. EM Aktiv and combined treatment applications are therefore recommended to boost c chlorophyll a, myo-inositol, TSS, propionic acid, TPC, kaempferol, and major lactones under autumn greenhouse conditions. To optimise both nutritional and sensory attributes, further research should investigate these interactions, focusing on long-term biofertiliser application and varying dosage levels. Future investigations integrating gene expression, proteomics, metabolomics, microbial ecology, employment of NMR and hyphenated chromatographic techniques to identify unknown polyphenols, sugars, and organic acids, are crucial to fully understand the complex interactions driving biofertiliser-induced metabolic changes and consequent plant responses.

## Figures and Tables

**Table 1:** Main and interaction effects on different spectrophotometric assays in lettuce cultivars.

Main Factors	TAC	TPC	Chl_a_	Chl_b_	Chl_(a+b)_	Total Car
***Cultivar***						
Kiribati	0.24 ± 0.02 a	217.05 ± 23.59 b	3.1 ± 0.23 c	3.4 ± 0.31 b	6.6 ± 0.39 c	0.8 ± 0.0 a
Murai	0.47 ± 0.03 c	271.58 ± 24.07 c	2.8 ± 0.17 bc	4.2 ± 0.39 c	7.4 ± 0.85 c	1.0 ± 0.17 a
Aquino	0.28 ± 0.03 a	127.11 ± 13.89 a	2.2 ± 0.25 ab	2.2 ± 0.17 a	4.4 ± 0.30 ab	0.8 ± 0.17 a
Gaugin	0.37 ± 0.03 b	347.54 ± 16.63 d	1.9 ± 0.25 a	2.1 ± 0.08 a	4.2 ± 0.08 ab	1.0 ± 0.0 a
Aleppo	0.26 ± 0.04 a	192.37 ± 11.44 b	2.1 ± 0.33 a	2.0 ± 0.0 a	3.8 ± 0.31 a	0.7 ± 0.25 a
Carmesi	0.52 ± 0.05 c	365.66 ± 35.42 d	2.3 ± 0.08 ab	2.5 ± 0.33 a	4.9 ± 0.17 b	1.8 ± 0.33 b
***Fertiliser***						
Control	0.37 ± 0.04	225.77 ± 13.05 a	2.1 ± 0.22 a	2.7 ± 0.21	5.1 ± 0.26	0.9 ± 0.17 ab
EM Aktiv	0.36 ± 0.04	267.78 ± 26.21 b	2.7 ± 0.26 b	2.8 ± 0.32	5.2 ± 0.43	0.8 ± 0.17 a
Vital Tricho	0.37 ± 0.02	250.61 ± 17.76 ab	2.6 ± 0.28 b	2.6 ± 0.17	5.4 ± 0.42	1.2 ± 0.17 b
EM Aktiv + Vital Tricho	0.33 ± 0.04	270.05 ± 26.35 b	2.2 ± 0.11 a	2.9 ± 0.17	5.1 ± 0.30	1.0 ± 0.11 ab
**Significance**						
Cultivar (C)	***	***	***	***	***	***
Fertiliser (F)	ns	*	***	ns	ns	*
**Interaction factors**						
C × F	***	***	***	***	***	ns

The data show the means (*n* = 3) ± SE. Values followed by the same letter are not significantly different at the 0.05% level of probability according to Tukey’s test. Groups of the same factors with no letters are not different from each other. Asterisks indicate significant differences at * *p* ≤ 0.05; ** *p* ≤ 0.01; *** *p* ≤ 0.001; ns, non-significant. TAC: total antioxidant capacity (mg AA eq g^−1^ FW), TPC: total phenolic content (µg GA eq g^−1^ FW), Chl_a_: chlorophyll a (µg g^−1^ FW), Chl_b_: chlorophyll b (µg g^−1^ FW), Chl_(a+b)_: chlorophyll (a + b) (µg g^−1^ FW), Total Car: total carotenoids (µg g^−1^ FW).

**Table 2:** Main and interaction effects on individual sugar content, total sweetness index, and total soluble solids in lettuce cultivars.

Main Factors	Myo	Glc	Fru	Suc	C1	C2	TSI	TSS
***Cultivar***								
Kiribati	2.43 ± 0.19 a	11.55 ± 0.86 a	12.34 ± 0.87 a	2.87 ± 0.30 a	2.19 ± 0.28 a	3.42 ± 0.35 b	30.15 ± 2.13 a	3.29 ± 0.39 a
Murai	3.38 ± 0.34 c	10.90 ± 1.23 a	12.29 ± 1.39 a	5.61 ± 0.82 bc	2.12 ± 0.27 a	6.49 ± 0.70 c	32.33 ± 3.45 a	3.42 ± 0.36 ab
Aquino	3.06 ± 0.38 abc	10.76 ± 1.09 a	12.29 ± 1.49 a	6.46 ± 0.48 c	2.58 ± 0.24 a	4.32 ± 0.51 b	33.06 ± 3.02 a	3.42 ± 0.31 ab
Gaugin	2.44 ± 0.34 a	10.11 ± 1.50 a	12.45 ± 1.55 a	4.84 ± 0.49 b	4.41 ± 0.47 b	2.92 ± 0.55 ab	32.65 ± 2.83 a	3.33 ± 0.31 a
Aleppo	2.61 ± 0.13 ab	11.38 ± 1.06 a	15.36 ± 1.37 ab	2.74 ± 0.30 a	2.17 ± 0.21 a	1.58 ± 0.26 a	34.43 ± 2.94 a	3.04 ± 0.34 a
Carmesi	3.22 ± 0.18 bc	15.33 ± 1.71 b	18.09 ± 1.96 b	8.95 ± 0.86 d	1.88 ± 0.15 a	7.41 ± 0.81 c	47.74 ± 4.48 b	4.17 ± 0.37 b
***Fertiliser***								
Control	2.80 ± 0.28 b	12.81 ± 1.43 b	15.22 ± 1.51 b	5.09 ± 0.67	2.33 ± 0.22 a	3.18 ± 0.36 a	37.66 ± 3.56 bc	3.08 ± 0.32 a
EM Aktiv	3.40 ± 0.36 c	12.83 ± 1.49 b	15.94 ± 1.46 b	5.62 ± 0.88	3.13 ± 0.46 b	4.99 ± 0.94 b	40.25 ± 3.27 c	3.42 ± 0.29 ab
Vital Tricho	2.90 ± 0.24 b	11.32 ± 1.12 ab	12.58 ± 1.42 a	5.58 ± 0.38	2.51 ± 0.15 a	5.79 ± 0.52 b	33.04 ± 2.88 ab	3.38 ± 0.33 ab
EM Aktiv + Vital Tricho	2.32 ± 0.16 a	9.73 ± 0.93 a	11.48 ± 1.36 a	4.68 ± 0.24	2.27 ± 0.23 a	3.47 ± 0.30 a	29.30 ± 2.87 a	3.90 ± 0.44 b
**Significance**								
Cultivar (C)	***	***	***	***	***	***	***	**
Fertiliser (F)	***	***	***	ns	***	***	***	**
**Interaction factors**								
C × F	***	***	***	***	***	***	***	***

The data show the means (*n* = 3) ± SE. Values followed by the same letter are not significantly different at the 0.05% level of probability according to Tukey’s test. Groups of the same factors with no letters are not different from each other. Asterisks indicate significant differences at * *p* ≤ 0.05; ** *p* ≤ 0.01; *** *p* ≤ 0.001; ns, non-significant. Myo: myo-inositol (mg g^−1^ FW), Glc: glucose (mg g^−1^ FW), Fru: fructose (mg g^−1^ FW), Suc: sucrose (mg g^−1^ FW), C1: unidentified compound **1** (mg arabinose eq g^−1^ FW), C2: unidentified compound **2** (mg maltose eq g^−1^ FW), TSI: total sweetness index, TSS: total soluble solids (°Brix).

**Table 3:** Main and interaction effects on organic acid content in lettuce cultivars.

Main Factors	Cit	Tar	C4	Mal	C3	Shik	Fum	Prop
***Cultivar***								
Kiribati	0.20 ± 0.02 bc	0.14 ± 0.016 d	0.05 ± 0.005 bc	3.77 ± 0.30 bc	0.18 ± 0.01 bc	39.58 ± 3.94 c	72.06 ± 4.72 d	0.70 ± 0.07 b
Murai	0.22 ± 0.01 c	0.08 ± 0.008 c	0.04 ± 0.002 bc	4.05 ± 0.41 c	0.12 ± 0.01 a	30.93 ± 1.03 ab	48.34 ± 4.66 bc	0.82 ± 0.08 b
Aquino	0.11 ± 0.01 a	0.07 ± 0.006 bc	0.05 ± 0.004 c	3.42 ± 0.29 abc	0.15 ± 0.02 ab	24.63 ± 2.16 a	41.41 ± 4.15 ab	0.41 ± 0.03 a
Gaugin	0.14 ± 0.01 a	0.05 ± 0.003 a	0.04 ± 0.003 b	3.51 ± 0.20 abc	0.12 ± 0.01 a	26.45 ± 2.43 a	42.14 ± 2.65 ab	1.44 ± 0.13 c
Aleppo	0.14 ± 0.01 a	0.07 ± 0.004 abc	0.04 ± 0.004 b	2.88 ± 0.25 a	0.18 ± 0.01 c	33.14 ± 2.88 bc	53.80 ± 4.28 c	0.72 ± 0.02 b
Carmesi	0.18 ± 0.02 b	0.06 ± 0.003 ab	0.03 ± 0.003 a	3.11 ± 0.29 ab	0.14 ± 0.01 a	28.63 ± 2.65 ab	35.97 ± 3.30 a	0.71 ± 0.07 b
***Fertiliser***								
Control	0.16 ± 0.01	0.07 ± 0.003	0.04 ± 0.003 a	3.42 ± 0.27	0.16 ± 0.01 b	30.15 ± 2.32 ab	48.13 ± 6.61	0.74 ± 0.08 a
EM Aktiv	0.16 ± 0.02	0.08 ± 0.007	0.05 ± 0.002 b	3.32 ± 0.24	0.18 ± 0.02 c	34.45 ± 3.52 b	49.82 ± 1.98	0.96 ± 0.06 b
Vital Tricho	0.17 ± 0.01	0.08 ± 0.008	0.04 ± 0.004 ab	3.42 ± 0.41	0.13 ± 0.01 a	28.84 ± 1.63 a	48.85 ± 4.60	0.76 ± 0.06 a
EM Aktiv + Vital Tricho	0.17 ± 0.01	0.08 ± 0.008	0.04 ± 0.003 ab	3.67 ± 0.25	0.14 ± 0.01 ab	28.81 ± 2.58 a	49.01 ± 2.65	0.74 ± 0.07 a
**Significance**								
Cultivar (C)	***	***	***	***	***	***	***	***
Fertiliser (F)	ns	ns	*	ns	***	*	ns	***
**Interaction factors**								
C × F	**	ns	***	**	**	***	***	***

The data show the means (*n* = 3) ± SE. Values followed by the same letter are not significantly different at the 0.05% level of probability according to Tukey’s test. Groups of the same factors with no letters are not different from each other. Asterisks indicate significant differences at * *p* ≤ 0.05; ** *p* ≤ 0.01; *** *p* ≤ 0.001; ns, non-significant. Cit: citric acid (mg g^−1^ FW), Tar: tartaric acid (mg g^−1^ FW), C4: unidentified compound **4** (mg oxalic acid eq g^−1^ FW), Mal: malic acid (mg g^−1^ FW), C3: unidentified compound **3** (mg formic acid eq g^−1^ FW), Shik: shikimic acid (µg g^−1^ FW), Fum: fumaric acid (µg g^−1^ FW), Prop: propionic acid (mg g^−1^ FW).

**Table 4:** Main and interaction effects on phenolic acid content in lettuce cultivars.

Main Factors	GA	CGA	Cfa	*p*CQA	Caf3G	Chic	CQA-Glc	CafG
***Cultivar***								
Kiribati	0.80 ± 0.09 a	0.45 ± 0.02 a	0.32 ± 0.03 b	0.07 ± 0.01 b	0.71 ± 0.05 c	2.74 ± 0.23 b	1.57 ± 0.13 c	0.42 ± 0.03 ab
Murai	1.71 ± 0.07 c	1.51 ± 0.13 d	0.31 ± 0.02 b	0.09 ± 0.01 c	0.70 ± 0.06 c	4.08 ± 0.42 c	2.01 ± 0.18 d	0.77 ± 0.05 e
Aquino	0.82 ± 0.12 a	0.37 ± 0.03 a	0.25 ± 0.02 a	0.06 ± 0.003 ab	0.57 ± 0.06 ab	2.77 ± 0.35 b	1.04 ± 0.10 a	0.33 ± 0.03 a
Gaugin	1.39 ± 0.11 b	0.96 ± 0.10 c	0.29 ± 0.02 ab	0.10 ± 0.01 c	0.52 ± 0.04 a	1.80 ± 0.07 a	1.22 ± 0.12 ab	0.55 ± 0.05 cd
Aleppo	1.04 ± 0.12 a	0.74 ± 0.07 b	0.33 ± 0.01 b	0.05 ± 0.004 a	0.68 ± 0.03 bc	3.34 ± 0.29 b	1.45 ± 0.13 bc	0.61 ± 0.06 d
Carmesi	1.52 ± 0.15 bc	1.10 ± 0.05 c	0.28 ± 0.02 ab	0.13 ± 0.01 d	0.72 ± 0.05 c	1.79 ± 0.17 a	1.29 ± 0.12 abc	0.46 ± 0.03 bc
***Fertiliser***								
Control	1.23 ± 0.15 ab	0.78 ± 0.04	0.29 ± 0.02	0.09 ± 0.01	0.61 ± 0.05	2.66 ± 0.28	1.31 ± 0.17	0.51 ± 0.05
EM Aktiv	1.25 ± 0.10 b	0.89 ± 0.05	0.31 ± 0.03	0.08 ± 0.01	0.68 ± 0.07	2.81 ± 0.23	1.47 ± 0.14	0.53 ± 0.05
Vital Tricho	1.05 ± 0.08 a	0.87 ± 0.08	0.30 ± 0.02	0.08 ± 0.01	0.65 ± 0.04	2.88 ± 0.21	1.50 ± 0.10	0.53 ± 0.04
EM Aktiv + Vital Tricho	1.33 ± 0.10 b	0.89 ± 0.10	0.29 ± 0.01	0.08 ± 0.01	0.65 ± 0.04	2.66 ± 0.29	1.44 ± 0.12	0.52 ± 0.03
**Significance**								
Cultivar (C)	***	***	***	***	***	***	***	***
Fertiliser (F)	**	ns	ns	ns	ns	ns	ns	ns
**Interaction factors**								
C × F	***	***	ns	***	ns	***	***	ns

The data show the means (*n* = 3) ± SE. Values followed by the same letter are not significantly different at the 0.05% level of probability according to Tukey’s test. Groups of the same factors with no letters are not different from each other. Asterisks indicate significant differences at * *p* ≤ 0.05; ** *p* ≤ 0.01; *** *p* ≤ 0.001; ns, non-significant. GA: gallic acid (µg g^−1^ FW), CGA: chlorogenic acid (µg g^−1^ FW), Cfa: caffeic acid (µg g^−1^ FW), *p*CQA: *p*-coumaroylquinic acid (µg coumaric acid eq g^−1^ FW), Caf3G: caffeic acid-3-glucoside (µg caffeic acid eq g^−1^ FW), Chic: chicoric acid (µg caffeic acid eq g^−1^ FW), CQA-Glc: caffeoylquinic acid glucoside (µg caffeic acid eq g^−1^ FW), CafG: caffeic acid glucuronide (µg caffeic acid eq g^−1^ FW).

**Table 5:** Main and interaction effects on flavonoid content in lettuce cultivars.

Main Factors	Q3G	Q3Glu	Q3AcGlc	Q3MalGlc	Q3MGlc7G	Kmp	K3MalG
***Cultivar***							
Kiribati	1.04 ± 0.08 ab	1.82 ± 0.16 b	0.34 ± 0.01 ab	1.30 ± 0.16 a	0.66 ± 0.05 ab	0.09 ± 0.006 ab	0.02 ± 0.004 a
Murai	4.02 ± 0.46 c	2.46 ± 0.18 b	0.63 ± 0.06 c	6.58 ± 0.29 b	1.05 ± 0.09 d	0.11 ± 0.008 c	0.14 ± 0.001 c
Aquino	0.55 ± 0.06 a	1.03 ± 0.11 a	0.28 ± 0.02 a	0.73 ± 0.07 a	0.51 ± 0.06 a	0.08 ± 0.005 a	0.00 ± 0.000 a
Gaugin	5.62 ± 0.44 d	2.00 ± 0.21 b	0.59 ± 0.04 c	6.63 ± 0.63 b	0.91 ± 0.06 cd	0.08 ± 0.006 a	0.07 ± 0.004 b
Aleppo	1.42 ± 0.12 b	1.00 ± 0.09 a	0.37 ± 0.02 b	1.89 ± 0.15 a	0.75 ± 0.06 bc	0.10 ± 0.010 bc	0.02 ± 0.006 a
Carmesi	5.16 ± 0.49 d	4.81 ± 0.59 c	0.83 ± 0.06 d	11.13 ± 1.26 c	1.96 ± 0.19 e	0.09 ± 0.007 ab	0.29 ± 0.030 d
***Fertiliser***							
Control	3.15 ± 0.28	2.01± 0.22	0.50 ± 0.04	4.90 ± 0.42	0.93 ± 0.09	0.08 ± 0.005 a	0.08 ± 0.008
EM Aktiv	2.71 ± 0.30	2.27 ± 0.25	0.50 ± 0.04	4.60 ± 0.50	0.99 ± 0.10	0.10 ± 0.009 b	0.09 ± 0.013
Vital Tricho	2.99 ± 0.29	2.14 ± 0.17	0.50 ± 0.02	4.50 ± 0.35	0.98 ± 0.07	0.09 ± 0.007 b	0.10 ± 0.009
EM Aktiv + Vital Tricho	3.01 ± 0.24	2.33 ± 0.24	0.53 ± 0.04	4.84 ± 0.44	1.00 ± 0.07	0.10 ± 0.007 b	0.09 ± 0.006
**Significance**							
Cultivar (C)	***	***	***	***	***	***	***
Fertiliser (F)	ns	ns	ns	ns	ns	***	ns
**Interaction factors**							
C × F	*	***	*	**	**	**	***

The data show the means (*n* = 3) ± SE. Values followed by the same letter are not significantly different at the 0.05% level of probability according to Tukey’s test. Groups of the same factors with no letters are not different from each other. Asterisks indicate significant differences at * *p* ≤ 0.05; ** *p* ≤ 0.01; *** *p* ≤ 0.001; ns, non-significant. Q3G: quercetin-3-glucoside (µg quercetin eq g^−1^ FW), Q3Glu: quercetin-3′-O-glucuronide (µg quercetin eq g^−1^ FW), Q3AcGlc: quercetin 3-O-(6″-acetyl-glucoside) (µg quercetin eq g^−1^ FW), Q3MalGlc: quercetin-3-O-(6″-O-malonyl)-glucoside (µg quercetin eq g^−1^ FW), Q3MGlc7G: quercetin 3-O-(6″-malonyl-glucoside) 7-O-glucoside (µg quercetin eq g^−1^ FW), Kmp: kaempferol (µg g^−1^ FW), K3MalG: kaempferol(6-O-malonyl) glucoside (µg kaempferol eq g^−1^ FW).

**Table 6:** Main and interaction effects on sesquiterpene lactones in lettuce cultivars.

Main Factors	Lc	Lc-ox	dLc	DHLc	DHLc-glu	Lp	Lp-ox	DHLp	DHLp-ox	STL
***Cultivar***										
Kiribati	1.30 ± 0.15 b	0.22 ± 0.004 c	0.08 ± 0.005 b	0.90 ± 0.10 d	0.91 ± 0.07 d	0.40 ± 0.04 b	0.90 ± 0.08 c	0.02 ± 0.002 ab	0.031 ± 0.002 d	4.79 ± 0.30 c
Murai	1.63 ± 0.15 bc	0.09 ± 0.007 b	0.04 ± 0.003 ab	0.48 ± 0.05 b	0.05 ± 0.001 a	0.32 ± 0.02 b	0.79 ± 0.05 bc	0.02 ± 0.002 a	0.015 ± 0.001 b	3.46 ± 0.22 b
Aquino	0.36 ± 0.05 a	0.04 ± 0.004 a	0.10 ± 0.011 b	0.42 ± 0.03 b	0.64 ± 0.08 c	0.20 ± 0.02 a	0.23 ± 0.03 a	0.03 ± 0.003 b	0.004 ± 0.001 a	2.10 ± 0.15 a
Gaugin	1.82 ± 0.24 c	0.04 ± 0.008 a	0.00 ± 0.00 a	0.74 ± 0.06 c	1.13 ± 0.06 e	0.33 ± 0.02 b	0.72 ± 0.05 b	0.04 ± 0.002 c	0.025 ± 0.002 cd	4.89 ± 0.26 c
Aleppo	0.59 ± 0.06 a	0.02 ± 0.003 a	0.17 ± 0.013 c	0.24 ± 0.03 a	0.13 ± 0.004 ab	0.39 ± 0.03 b	0.89 ± 0.05 c	0.02 ± 0.002 a	0.021 ± 0.001 bc	2.53 ± 0.13 a
Carmesi	1.52 ± 0.14 bc	0.29 ± 0.023 d	0.44 ± 0.058 d	0.86 ± 0.06 cd	0.23 ± 0.006 b	0.64 ± 0.08 c	2.28 ± 0.12 d	0.05 ± 0.005 c	0.075 ± 0.007 e	6.38 ± 0.28 d
***Fertiliser***										
Control	1.06 ± 0.15 a	0.27 ± 0.015 c	0.07 ± 0.003 a	0.52 ± 0.05 a	0.52 ± 0.04 ab	0.36 ± 0.04 a	0.80 ± 0.05 a	0.03 ± 0.003	0.025 ± 0.001 a	3.68 ± 0.23 a
EM Aktiv	1.02 ± 0.10 a	0.10 ± 0.009 b	0.20 ± 0.029 c	0.69 ± 0.07 b	0.52 ± 0.02 ab	0.45 ± 0.06 b	1.18 ± 0.08 c	0.03 ± 0.002	0.028 ± 0.003 ab	4.22 ± 0.14 b
Vital Tricho	1.34 ± 0.11 b	0.07 ± 0.004 a	0.12 ± 0.006 ab	0.61 ± 0.05 ab	0.58 ± 0.04 b	0.34 ± 0.02 a	0.90 ± 0.04 ab	0.03 ± 0.004	0.029 ± 0.002 ab	4.07 ± 0.19 ab
EM Aktiv + Vital Tricho	1.40 ± 0.16 b	0.05 ± 0.006 a	0.16 ± 0.023 bc	0.61 ± 0.06 ab	0.44 ± 0.04 a	0.36 ± 0.02 a	1.00 ± 0.08 b	0.03 ± 0.002	0.032 ± 0.003 b	4.14 ± 0.34 b
**Significance**										
Cultivar (C)	***	***	***	***	***	***	***	***	***	***
Fertiliser (F)	***	***	***	**	***	**	***	ns	**	**
**Interaction factors**										
C × F	***	***	***	***	***	***	***	***	***	***

The data show the means (*n* = 3) ± SE. Values followed by the same letter are not significantly different at the 0.05% level of probability according to Tukey’s test. Groups of the same factors with no letters are not different from each other. Asterisks indicate significant differences at * *p* ≤ 0.05; ** *p* ≤ 0.01; *** *p* ≤ 0.001; ns, non-significant. All results are expressed as peak areas proportional to the actual amounts of compounds detected in the sample. Lc: lactucin, Lc-ox: lactucin-15-oxalate, dLc: 8-deoxylactucin, DHLc: 11β,13-dihydrolactucin, DHLc-glu: 11β,13-dihydrolactucin-15-glucoside, Lp: lactucopicrin, Lp-ox: lactucopicrin-15-oxalate, DHLp: 11β,13-dihydrolactucopicrin, DHLp-ox: 11β,13-dihydrolactucopicrin-15-oxalate, STL: total lactones.

**Table 7:** Main and interaction effects on the overall taste attribute in lettuce cultivars.

Main Factors	OT
***Cultivar***	
Kiribati	2.56 ± 0.21 a
Murai	2.90 ± 0.21 ab
Aquino	3.08 ± 0.25 b
Gaugin	2.58 ± 0.28 a
Aleppo	2.54 ± 0.17 a
Carmesi	2.79 ± 0.19 ab
***Fertiliser***	
Control	2.76 ± 0.21 ab
EM Aktiv	2.75 ± 0.24 ab
Vital Tricho	2.54 ± 0.21 a
EM Aktiv + Vital Tricho	2.92 ± 0.21 b
**Significance**	
Cultivar (C)	***
Fertiliser (F)	*
**Interaction factors**	
C × F	***

The data show the means (*n* = 12) ± SE. Values followed by the same letter are not significantly different at the 0.05% level of probability according to Tukey’s test. Groups of the same factors with no letters are not different from each other. Asterisks indicate significant differences at * *p* ≤ 0.05; ** *p* ≤ 0.01; *** *p* ≤ 0.001; ns, non-significant. OT: overall taste.

## Data Availability

All new research data were presented in this contribution.

## Supplementary Materials

The following supporting information can be downloaded at: https://www.mdpi.com/article/10.3390/plants14243864/s1, Figure S1: The heat map correlation matrix of tested parameters in lettuce and Table S1: Pearson correlation analysis of tested parameters in lettuce.

## Abbreviations

The following abbreviations are used in this manuscript:

EM	Effective microorganisms
TAC	Total antioxidant capacity
TPC	Total phenolic content
TSS	Total soluble solids
Chl_a_	Chlorophyll a
Chl_b_	Chlorophyll b
Chl_(a + b)_	Chlorophyll (a+b)
Total Car	Total carotenoids
Lp	Lactucopicrin
Lc	Lactucin
DHLc	11β,13-dihydrolactucin
Lp-ox	Lactucopicrin-15-oxalate
dLc	8-deoxylactucin
DHLc-glu	11β,13-dihydrolactucin-15-glucoside
Lc-ox	Lactucin-15-oxalate
DHLp-ox	11β,13-dihydrolactucopicrin-15-oxalate
DHLp	11β,13-dihydrolactucopicrin
STL	Total lactones
Myo	Myo-inositol
Glc	Glucose
Fru	Fructose
Suc	Sucrose
Arabinose eq-C1	Unidentified compound **1** (arabinose equivalents)
Maltose eq-C2	Unidentified compound **2** (maltose equivalents)
TSI	Total sweetness index
Cit	Citric acid
Tar	Tartaric acid
Mal	Malic acid
Fum	Fumaric acid
Prop	Propionic acid
Shik	Shikimic acid
Formic eq-C3	Unidentified compound **3** (formic acid equivalents)
Oxalic eq-C4	Unidentified compound **4** (oxalic acid equivalents)
CGA	Chlorogenic acid
Cfa	Caffeic acid
Kmp	Kaempferol
GA	Gallic acid
*p*CQA	*p*-coumaroylquinic acid
K3MalG	Kaempferol(6-O-malonyl) glucoside
Q3Glu	Quercetin-3′-O-glucuronide
Q3MalGlc	Quercetin-3-O-(6″-O-malonyl)-glucoside
Q3AcGlc	Quercetin 3-O-(6″-acetyl-glucoside)
Q3MGlc7G	Quercetin 3-O-(6″-malonyl-glucoside) 7-O-glucoside
Q3G	Quercetin-3-glucoside
CQA-Glc	Caffeoylquinic acid glucoside
Caf3G	Caffeic acid-3-glucoside
CafG	Caffeic acid glucuronide
Chic	Chicoric acid
OT	Overall taste
AMF	Arbuscular mycorrhizal fungi
UDP-glucose	Uridine diphosphate glucose
PAL	Phenylalanine ammonia lyase
DAD	Diode Array Detector
ESI	Electrospray ionisation
HRP	Horseradish peroxidase
ABTS	2,2′-azino-bis-(3-ethylbenzothiazoline-6-sulfonic) acid
AA	Ascorbic acid
