# Supplementary material for: Phytochemical Composition and Overall Taste Modulation in Lettuce: Combination of Cultivar and Biofertiliser
Source: Plants (Basel). 2025 Dec 18;14(24):3864. doi: 10.3390/plants14243864 (PMC12737260; doi:10.3390/plants14243864)
Supplement: Supplementary file 1 [file plants-14-03864-s001.zip › Supplementary Figure S1.pdf]

Figure S1. The heat map correlation matrix of tested parameters in lettuce

	TAC	TPC	TSS	Chl_a	Chl_b	$Chl_{(a+b)}$	Total Car	Lp	Lc	DHLc	Lp−ox	dLc	DHLc−glu	Lc−ox	DHLp−ox	DHLp
TAC	1															
TPC	0.40	1														
TSS	0.25	0.06	1													
Chl_a	−0.02	0.06	0.06	1												
Chl_b	0.16	0.09	0.22	0.44	1											
$Chl_{(a+b)}$	0.16	0.12	0.16	0.66	0.85	1										
Total Car	0.34	0.40	0.12	0.01	−0.07	−0.06	1									
Lp	0.16	0.34	0.02	0.08	−0.10	−0.06	0.22	1								
Lc	0.35	0.61	0.10	0.23	0.24	0.33	0.29	0.34	1							
DHLc	0.17	0.35	0.32	0.34	0.18	0.28	0.24	0.34	0.58	1						
Lp−ox	0.32	0.52	0.21	0.17	−0.01	0.05	0.43	0.77	0.48	0.48	1					
dLc	0.28	0.21	0.26	0.02	−0.21	−0.18	0.36	0.68	0.11	0.33	0.83	1				
DHLc−glu	−0.21	0.06	−0.15	−0.03	−0.17	−0.10	−0.13	0.05	0.33	0.43	−0.06	−0.25	1			
Lc−ox	0.08	0.07	−0.02	0.11	0.05	0.06	0.22	0.21	−0.16	0.04	0.23	0.15	−0.26	1		
DHLp−ox	0.39	0.53	0.29	0.07	−0.05	−0.02	0.54	0.62	0.40	0.55	0.81	0.74	−0.12	0.35	1	
DHLp	0.17	0.49	0.15	−0.10	−0.29	−0.20	0.34	0.53	0.46	0.47	0.57	0.39	0.37	0.15	0.61	1
STL	0.30	0.58	0.16	0.22	0.05	0.15	0.38	0.70	0.79	0.76	0.84	0.55	0.38	0.12	0.72	0.69
Myo	0.14	−0.03	0.07	−0.02	−0.03	−0.03	0.16	0.08	−0.22	−0.08	0.10	0.21	−0.29	0.08	−0.004	−0.02
Glc	0.03	0.10	0.17	−0.01	0.05	0.07	0.18	0.19	−0.08	0.17	0.29	0.30	−0.15	0.20	0.27	0.15
Fru	0.11	0.12	0.15	−0.15	−0.09	−0.13	0.13	0.21	−0.24	0.01	0.27	0.32	−0.26	0.25	0.27	0.10
Suc	0.47	0.25	0.33	−0.02	−0.10	−0.06	0.53	0.17	0.000	0.12	0.32	0.37	−0.15	0.29	0.38	0.37
Arabinose eq−C1	−0.11	0.21	−0.06	−0.09	−0.13	−0.07	−0.16	−0.25	−0.10	0.02	−0.22	−0.26	0.19	−0.11	−0.25	−0.11
Maltose eq− C2	0.43	0.22	0.32	0.18	0.19	0.25	0.32	0.11	0.03	0.01	0.32	0.26	−0.25	0.28	0.24	0.05
TSI	0.20	0.20	0.22	−0.11	−0.08	−0.09	0.27	0.22	−0.19	0.11	0.33	0.37	−0.23	0.28	0.35	0.21
Cit	0.36	0.28	0.14	0.38	0.64	0.60	0.15	0.09	0.44	0.28	0.22	0.01	−0.15	−0.03	0.17	−0.17
Tar	−0.34	−0.24	−0.07	0.46	0.37	0.42	−0.26	−0.13	−0.02	0.26	−0.15	−0.22	0.16	−0.05	−0.14	−0.34
Mal	0.11	0.10	0.04	0.13	0.30	0.27	0.04	−0.22	0.20	0.16	−0.11	−0.19	0.07	−0.12	−0.22	−0.30
Fum	−0.31	−0.19	−0.07	0.38	0.23	0.34	−0.29	−0.19	−0.09	0.13	−0.16	−0.24	0.10	−0.01	−0.19	−0.46
Prop	0.02	0.48	−0.09	−0.10	−0.06	−0.06	0.10	−0.17	0.24	0.19	−0.07	−0.26	0.20	−0.15	−0.01	0.07
Shik	−0.23	0.05	−0.08	0.30	0.23	0.34	−0.17	−0.01	−0.08	0.19	0.05	−0.01	−0.04	−0.04	0.000	−0.28
Formic eq−C3	−0.21	−0.25	−0.26	0.01	−0.19	−0.19	−0.25	−0.01	−0.44	−0.10	−0.04	0.07	−0.02	0.05	−0.09	−0.23
Oxalic eq−C4	−0.29	−0.37	−0.26	−0.07	−0.22	−0.20	−0.09	−0.32	−0.25	−0.22	−0.46	−0.29	0.03	−0.13	−0.45	−0.29
CGA	0.56	0.58	0.02	0.08	0.21	0.22	0.26	0.31	0.64	0.06	0.45	0.20	−0.23	−0.08	0.26	0.22
Cfa	−0.09	0.04	−0.24	0.30	0.000	0.05	−0.06	0.24	0.15	0.001	0.13	0.05	−0.05	−0.02	0.04	−0.22
Kmp	0.17	0.11	0.13	0.30	0.42	0.39	−0.11	0.14	0.13	−0.06	0.13	0.13	−0.38	−0.22	0.01	−0.25
GA	0.48	0.43	0.15	−0.14	0.25	0.10	0.36	0.26	0.43	0.09	0.28	0.15	−0.24	0.02	0.29	0.18
pCQA	0.52	0.70	0.21	−0.11	0.02	0.02	0.54	0.41	0.55	0.39	0.58	0.38	0.07	0.25	0.58	0.52
K3MalG	0.67	0.65	0.30	0.08	0.14	0.15	0.65	0.45	0.52	0.41	0.74	0.61	−0.29	0.20	0.77	0.49
Q3Glu	0.56	0.68	0.28	0.08	0.13	0.14	0.52	0.45	0.52	0.48	0.69	0.56	−0.10	0.14	0.75	0.51
Q3MalGlc	0.67	0.78	0.22	−0.06	0.04	0.05	0.56	0.41	0.60	0.33	0.63	0.43	−0.15	0.19	0.67	0.54
Q3AcGlc	0.66	0.77	0.21	−0.01	0.13	0.12	0.54	0.42	0.61	0.33	0.63	0.42	−0.18	0.15	0.65	0.46
Q3MGlc7G	0.59	0.69	0.24	0.03	0.04	0.05	0.59	0.53	0.50	0.36	0.77	0.64	−0.21	0.18	0.78	0.52
Q3G	0.55	0.75	0.16	−0.15	−0.01	0.01	0.48	0.26	0.65	0.31	0.42	0.17	0.02	0.12	0.47	0.47
CQA−Glc	0.22	0.18	−0.05	0.48	0.53	0.56	−0.01	0.01	0.38	0.07	0.12	−0.13	−0.20	−0.14	−0.01	−0.25
Caf3G	0.12	0.13	−0.09	0.32	0.12	0.16	0.19	0.38	0.10	0.05	0.37	0.39	−0.27	0.07	0.30	−0.01
CafG	0.28	0.28	−0.14	0.05	0.20	0.15	−0.003	0.14	0.38	−0.21	0.11	−0.01	−0.27	−0.17	−0.10	−0.18
Chic	0.02	−0.26	−0.23	0.19	0.25	0.19	−0.29	−0.11	0.02	−0.40	−0.15	−0.21	−0.24	−0.14	−0.36	−0.32
OT	0.11	−0.12	0.08	−0.14	−0.03	0.002	−0.11	−0.05	−0.04	0.14	−0.05	0.05	0.01	−0.06	−0.06	0.01

	STL	Myo	Glc	Fru	Suc	Arabinose eq−C1	Maltose eq− C2	TSI	Cit	Tar	Mal	Fum	Prop	Shik	Formic eq−C3	Oxalic eq−C4
STL	1															
Myo	−0.01	1														
Glc	0.15	0.50	1													
Fru	0.03	0.46	0.85	1												
Suc	0.12	0.49	0.29	0.28	1											
Arabinose eq−C1	−0.14	0.23	0.14	0.14	0.04	1										
Maltose eq− C2	0.16	0.53	0.38	0.37	0.65	0.14	1									
TSI	0.11	0.59	0.89	0.93	0.52	0.22	0.50	1								
Cit	0.27	0.03	0.02	−0.08	−0.02	−0.15	0.21	−0.07	1							
Tar	−0.03	−0.11	0.02	−0.11	−0.40	−0.08	−0.05	−0.19	0.42	1						
Mal	0.02	0.18	0.03	−0.09	0.003	0.31	0.23	−0.03	0.53	0.26	1					
Fum	−0.10	−0.11	0.06	0.02	−0.41	0.14	−0.04	−0.07	0.32	0.65	0.45	1				
Prop	0.09	0.10	0.08	0.08	0.01	0.72	0.01	0.15	0.06	−0.13	0.27	0.11	1			
Shik	0.01	0.15	0.38	0.33	−0.24	0.29	0.05	0.27	0.33	0.54	0.29	0.66	0.27	1		
Formic eq−C3	−0.20	0.12	0.17	0.34	−0.23	0.07	−0.18	0.22	−0.05	0.33	−0.01	0.47	0.01	0.50	1	
Oxalic eq−C4	−0.39	0.10	−0.17	−0.18	−0.12	−0.14	−0.17	−0.22	−0.13	0.23	0.06	0.04	−0.12	0.003	0.12	1
CGA	0.42	0.14	0.000	−0.03	0.17	−0.13	0.24	0.02	0.40	−0.33	0.12	−0.27	0.18	−0.12	−0.38	−0.24
Cfa	0.12	0.06	−0.03	0.01	−0.27	−0.04	−0.05	−0.10	0.24	0.33	0.02	0.27	0.08	0.32	0.24	0.14
Kmp	−0.002	0.17	0.02	0.02	−0.18	−0.08	0.11	−0.05	0.44	0.23	0.23	0.17	−0.10	0.20	0.05	−0.02
GA	0.28	0.13	0.004	0.03	0.20	−0.07	0.06	0.08	0.33	−0.38	0.03	−0.45	0.22	−0.22	−0.34	−0.18
pCQA	0.64	0.10	0.21	0.20	0.47	0.10	0.40	0.30	0.19	−0.36	0.07	−0.40	0.26	−0.19	−0.39	−0.45
K3MalG	0.63	0.17	0.24	0.23	0.50	−0.24	0.40	0.33	0.37	−0.32	−0.01	−0.38	0.03	−0.10	−0.27	−0.41
Q3Glu	0.66	0.13	0.31	0.26	0.42	−0.14	0.34	0.35	0.34	−0.16	−0.003	−0.30	0.05	−0.02	−0.22	−0.40
Q3MalGlc	0.61	0.10	0.18	0.19	0.45	−0.06	0.31	0.29	0.25	−0.46	−0.02	−0.46	0.21	−0.20	−0.37	−0.38
Q3AcGlc	0.60	0.08	0.15	0.17	0.40	−0.06	0.31	0.26	0.33	−0.39	−0.01	−0.42	0.20	−0.15	−0.36	−0.39
Q3MGlc7G	0.65	0.10	0.24	0.25	0.43	−0.20	0.32	0.33	0.27	−0.33	−0.09	−0.39	0.02	−0.10	−0.24	−0.42
Q3G	0.53	0.01	0.07	0.09	0.34	0.13	0.21	0.18	0.14	−0.50	0.02	−0.42	0.43	−0.21	−0.47	−0.27
CQA−Glc	0.13	0.02	0.01	−0.11	−0.18	−0.13	0.18	−0.11	0.54	0.30	0.24	0.29	0.05	0.23	−0.07	−0.19
Caf3G	0.21	0.22	0.13	0.11	0.04	−0.28	0.16	0.07	0.44	0.34	0.05	0.16	−0.15	0.33	0.19	0.05
CafG	0.07	0.10	−0.09	−0.04	−0.14	−0.09	0.05	−0.10	0.36	−0.13	0.15	−0.02	0.18	0.04	−0.18	−0.01
Chic	−0.23	0.05	−0.20	−0.25	−0.26	−0.34	−0.04	−0.32	0.22	0.13	0.003	0.10	−0.31	−0.02	0.06	0.07
OT	−0.02	0.12	0.24	0.13	0.14	0.05	0.06	0.20	−0.16	0.01	0.08	0.01	−0.08	−0.03	0.03	−0.01

	CGA	Cfa	Kmp	GA	pCQA	K3MalG	Q3Glu	Q3MalGlc	Q3AcGlc	Q3MGlc7G	Q3G	CQA−Glc	Caf3G	CafG	Chic	OT
CGA	1															
Cfa	0.19	1														
Kmp	0.34	0.35	1													
GA	0.68	0.09	0.19	1												
pCQA	0.52	−0.09	−0.11	0.51	1											
K3MalG	0.62	−0.08	0.13	0.57	0.76	1										
Q3Glu	0.54	−0.04	0.11	0.48	0.80	0.88	1									
Q3MalGlc	0.71	−0.05	0.07	0.66	0.86	0.90	0.88	1								
Q3AcGlc	0.73	0.05	0.12	0.69	0.83	0.88	0.87	0.97	1							
Q3MGlc7G	0.59	0.01	0.12	0.53	0.78	0.93	0.92	0.91	0.91	1						
Q3G	0.67	0.003	−0.02	0.66	0.79	0.68	0.65	0.89	0.87	0.70	1					
CQA−Glc	0.52	0.48	0.43	0.23	0.02	0.18	0.19	0.15	0.28	0.16	0.11	1				
Caf3G	0.31	0.67	0.37	0.22	0.12	0.30	0.37	0.20	0.28	0.38	−0.01	0.46	1			
CafG	0.75	0.43	0.34	0.50	0.14	0.19	0.14	0.33	0.40	0.22	0.40	0.61	0.34	1		
Chic	0.36	0.33	0.30	0.04	−0.29	−0.13	−0.20	−0.19	−0.14	−0.18	−0.28	0.58	0.30	0.51	1	
OT	0.07	−0.14	−0.02	0.02	0.04	−0.03	0.09	0.01	−0.01	−0.03	−0.02	0.06	−0.12	−0.07	0.10	1

Each cell in the correlation matrix is represented by a Pearson's correlation coefficient (r). Their correlated levels were represented with blue (strong positive relationship) to red (strong negative relationship).

TAC: total antioxidant capacity, TPC: total phenolic content, TSS: total soluble solids, Chla: chlorophyll a, Chlb: chlorophyll b, Chl(a+b): chlorophyll (a+b), Total Car: total carotenoids, Lp: lactucopicrin, Lc: lactucin, DHLc: 11β,13-dihydrolactucin, Lp-ox: lactucopicrin-15-oxalate, dLc: 8-deoxylactucin, DHLc-glu: 11β,13-dihydrolactucin-15-glucoside, Lc-ox: lactucin-15-oxalate, DHLp-ox: 11β,13-dihydrolactucopicrin-15-oxalate, DHLp: 11β,13-dihydrolactucopicrin, STL: total lactones, Myo: myo-inositol, Glc: glucose, Fru: fructose, Suc: sucrose, C1: unidentified compound 1 (arabinose equivalents), C2: unidentified compound 2 (maltose equivalents), TSI: total sweetness index, Cit: citric acid, Tar: tartaric acid, Mal: malic acid, Fum: fumaric acid, Prop: propionic acid, Shik: shikimic acid, C3: unidentified compound 3 (formic acid equivalents), C4: unidentified compound 4 (oxalic acid equivalents), CGA: chlorogenic acid, Cfa: caffeic acid, Kmp: kaempferol, GA: gallic acid, pCQA: *p*-coumaroylquinic acid, K3MalG: kaempferol(6-O-malonyl) glucoside, Q3Glu: quercetin-3′-O-glucuronide, Q3MalGlc: quercetin-3-O-(6″-O-malonyl)-glucoside, Q3AcGlc: quercetin 3-O-(6″-acetyl-glucoside), Q3MGlc7G: quercetin 3-O-(6"-malonyl-glucoside) 7-O-glucoside, Q3G: quercetin-3-glucoside, CQA-Glc: caffeoylquinic acid glucoside, Caf3G: caffeic acid-3-glucoside, CafG: caffeic acid glucuronide, Chic: chicoric acid, OT: overall taste.
