# Supplementary material for: Phytochemical Composition and Overall Taste Modulation in Lettuce: Combination of Cultivar and Biofertiliser
Source: Plants (Basel). 2025 Dec 18;14(24):3864. doi: 10.3390/plants14243864 (PMC12737260; doi:10.3390/plants14243864)
Supplement: Supplementary file 1 [file plants-14-03864-s001.zip › Supplementary Table S1.pdf]

Table S1. Pearson correlation analysis of tested parameters in lettuce

	TAC	TPC	TSS	Chl_a	Chl_b	$Chl_{(a+b)}$	Total Car	Lp	Lc	DHLc	Lp−ox	dLc	DHLc−glu	Lc−ox	DHLp−ox	DHLp	STL	Myo	Glc	Fru	Suc
TAC	1																				
TPC	0.40**	1																			
TSS	0.25*	0.06	1																		
Chl_a	−0.02	0.06	0.06	1																	
Chl_b	0.16	0.09	0.22	0.44**	1																
$Chl_{(a+b)}$	0.16	0.12	0.16	0.66**	0.85**	1															
Total Car	0.34**	0.40**	0.12	0.01	−0.07	−0.06	1														
Lp	0.16	0.34**	0.02	0.08	−0.10	−0.06	0.22	1													
Lc	0.35**	0.61**	0.10	0.23	0.24*	0.33**	0.29*	0.34**	1												
DHLc	0.17	0.35**	0.32**	0.34**	0.18	0.28*	0.24*	0.34**	0.58**	1											
Lp−ox	0.32**	0.52**	0.21	0.17	−0.01	0.05	0.43**	0.77**	0.48**	0.48**	1										
dLc	0.28*	0.21	0.26*	0.02	−0.21	−0.18	0.36**	0.68**	0.11	0.33**	0.83**	1									
DHLc−glu	−0.21	0.06	−0.15	−0.03	−0.17	−0.10	−0.13	0.05	0.33**	0.43**	−0.06	−0.25*	1								
Lc−ox	0.08	0.07	−0.02	0.11	0.05	0.06	0.22	0.21	−0.16	0.04	0.23	0.15	−0.26*	1							
DHLp−ox	0.39**	0.53**	0.29*	0.07	−0.05	−0.02	0.54**	0.62**	0.40**	0.55**	0.81**	0.74**	−0.12	0.35**	1						
DHLp	0.17	0.49**	0.15	−0.10	−0.29*	−0.20	0.34**	0.53**	0.46**	0.47**	0.57**	0.39**	0.37**	0.15	0.61**	1					
STL	0.30*	0.58**	0.16	0.22	0.05	0.15	0.38**	0.70**	0.79**	0.76**	0.84**	0.55**	0.38**	0.12	0.72**	0.69**	1				
Myo	0.14	−0.03	0.07	−0.02	−0.03	−0.03	0.16	0.08	−0.22	−0.08	0.10	0.21	−0.29*	0.08	−0.004	−0.02	−0.01	1			
Glc	0.03	0.10	0.17	−0.01	0.05	0.07	0.18	0.19	−0.08	0.17	0.29*	0.30*	−0.15	0.20	0.27*	0.15	0.15	0.50**	1		
Fru	0.11	0.12	0.15	−0.15	−0.09	−0.13	0.13	0.21	−0.24*	0.01	0.27*	0.32**	−0.26*	0.25*	0.27*	0.10	0.03	0.46**	0.85**	1	
Suc	0.47**	0.25*	0.33**	−0.02	−0.10	−0.06	0.53**	0.17	0.000	0.12	0.32**	0.37**	−0.15	0.29*	0.38**	0.37**	0.12	0.49**	0.29*	0.28*	1
Arabinose eq−C1	−0.11	0.21	−0.06	−0.09	−0.13	−0.07	−0.16	−0.25*	−0.10	0.02	−0.22	−0.26*	0.19	−0.11	−0.25*	−0.11	−0.14	0.23*	0.14	0.14	0.04
Maltose eq− C2	0.43**	0.22	0.32**	0.18	0.19	0.25*	0.32**	0.11	0.03	0.01	0.32**	0.26*	−0.25*	0.28*	0.24*	0.05	0.16	0.53**	0.38**	0.37**	0.65**
TSI	0.20	0.20	0.22	−0.11	−0.08	−0.09	0.27*	0.22	−0.19	0.11	0.33**	0.37**	−0.23*	0.28*	0.35**	0.21	0.11	0.59**	0.89**	0.93**	0.52**
Cit	0.36**	0.28*	0.14	0.38**	0.64**	0.60**	0.15	0.09	0.44**	0.28*	0.22	0.01	−0.15	−0.03	0.17	−0.17	0.27*	0.03	0.02	−0.08	−0.02
Tar	−0.34**	−0.24*	−0.07	0.46**	0.37**	0.42**	−0.26*	−0.13	−0.02	0.26*	−0.15	−0.22	0.16	−0.05	−0.14	−0.34**	−0.03	−0.11	0.02	−0.11	−0.40**
Mal	0.11	0.10	0.04	0.13	0.30*	0.27*	0.04	−0.22	0.20	0.16	−0.11	−0.19	0.07	−0.12	−0.22	−0.30*	0.02	0.18	0.03	−0.09	0.003
Fum	−0.31**	−0.19	−0.07	0.38**	0.23	0.34**	−0.29*	−0.19	−0.09	0.13	−0.16	−0.24*	0.10	−0.01	−0.19	−0.46**	−0.10	−0.11	0.06	0.02	−0.41**
Prop	0.02	0.48**	−0.09	−0.10	−0.06	−0.06	0.10	−0.17	0.24*	0.19	−0.07	−0.26*	0.20	−0.15	−0.01	0.07	0.09	0.10	0.08	0.08	0.01
Shik	−0.23	0.05	−0.08	0.30*	0.23	0.34**	−0.17	−0.01	−0.08	0.19	0.05	−0.01	−0.04	−0.04	0.000	−0.28*	0.01	0.15	0.38**	0.33**	−0.24*
Formic eq−C3	−0.21	−0.25*	−0.26*	0.01	−0.19	−0.19	−0.25*	−0.01	−0.44**	−0.10	−0.04	0.07	−0.02	0.05	−0.09	−0.23	−0.20	0.12	0.17	0.34**	−0.23*
Oxalic eq−C4	−0.29*	−0.37**	−0.26*	−0.07	−0.22	−0.20	−0.09	−0.32**	−0.25*	−0.22	−0.46**	−0.29*	0.03	−0.13	−0.45**	−0.29*	−0.39**	0.10	−0.17	−0.18	−0.12
CGA	0.56**	0.58**	0.02	0.08	0.21	0.22	0.26*	0.31**	0.64**	0.06	0.45**	0.20	−0.23*	−0.08	0.26*	0.22	0.42**	0.14	0.000	−0.03	0.17
Cfa	−0.09	0.04	−0.24*	0.30*	0.000	0.05	−0.06	0.24*	0.15	0.001	0.13	0.05	−0.05	−0.02	0.04	−0.22	0.12	0.06	−0.03	0.01	−0.27*
Kmp	0.17	0.11	0.13	0.30*	0.42**	0.39**	−0.11	0.14	0.13	−0.06	0.13	0.13	−0.38**	−0.22	0.01	−0.25*	−0.002	0.17	0.02	0.02	−0.18
GA	0.48**	0.43**	0.15	−0.14	0.25*	0.10	0.36**	0.26*	0.43**	0.09	0.28*	0.15	−0.24*	0.02	0.29*	0.18	0.28*	0.13	0.004	0.03	0.20
pCQA	0.52**	0.70**	0.21	−0.11	0.02	0.02	0.54**	0.41**	0.55**	0.39**	0.58**	0.38**	0.07	0.25*	0.58**	0.52**	0.64**	0.10	0.21	0.20	0.47**
K3MalG	0.67**	0.65**	0.30*	0.08	0.14	0.15	0.65**	0.45**	0.52**	0.41**	0.74**	0.61**	−0.29*	0.20	0.77**	0.49**	0.63**	0.17	0.24*	0.23	0.50**
Q3Glu	0.56**	0.68**	0.28*	0.08	0.13	0.14	0.52**	0.45**	0.52**	0.48**	0.69**	0.56**	−0.10	0.14	0.75**	0.51**	0.66**	0.13	0.31**	0.26*	0.42**
Q3MalGlc	0.67**	0.78**	0.22	−0.06	0.04	0.05	0.56**	0.41**	0.60**	0.33**	0.63**	0.43**	−0.15	0.19	0.67**	0.54**	0.61**	0.10	0.18	0.19	0.45**
Q3AcGlc	0.66**	0.77**	0.21	−0.01	0.13	0.12	0.54**	0.42**	0.61**	0.33**	0.63**	0.42**	−0.18	0.15	0.65**	0.46**	0.60**	0.08	0.15	0.17	0.40**
Q3MGlc7G	0.59**	0.69**	0.24*	0.03	0.04	0.05	0.59**	0.53**	0.50**	0.36**	0.77**	0.64**	−0.21	0.18	0.78**	0.52**	0.65**	0.10	0.24*	0.25*	0.43**
Q3G	0.55**	0.75**	0.16	−0.15	−0.01	0.01	0.48**	0.26*	0.65**	0.31**	0.42**	0.17	0.02	0.12	0.47**	0.47**	0.53**	0.01	0.07	0.09	0.34**
CQA−Glc	0.22	0.18	−0.05	0.48**	0.53**	0.56**	−0.01	−0.01	0.38**	0.07	0.12	−0.13	−0.20	−0.14	−0.01	−0.25*	0.13	0.02	0.01	−0.11	−0.18
Caf3G	0.12	0.13	−0.09	0.32**	0.12	0.16	0.19	0.38**	0.10	0.05	0.37**	0.39**	−0.27*	0.07	0.30*	−0.01	0.21	0.22	0.13	0.11	0.04
CafG	0.28*	0.28*	−0.14	0.05	0.20	0.15	−0.003	0.14	0.38**	−0.21	0.11	−0.01	−0.27*	−0.17	−0.10	−0.18	0.07	0.10	−0.09	−0.04	−0.14
Chic	0.02	−0.26*	−0.23*	0.19	0.25*	0.19	−0.29*	−0.11	0.02	−0.40**	−0.15	−0.21	−0.24*	−0.14	−0.36**	−0.32**	−0.23	0.05	−0.20	−0.25*	−0.26*
OT	0.11	−0.12	0.08	−0.14	−0.03	0.002	−0.11	−0.05	−0.04	0.14	−0.05	0.05	0.01	−0.06	−0.06	0.01	−0.02	0.12	0.24*	0.13	0.14

	Arabinose eq−C1	Maltose eq− C2	TSI	Cit	Tar	Mal	Fum	Prop	Shik	Formic eq−C3	Oxalic eq−C4	CGA	Cfa	Kmp	GA
Arabinose eq−C1	1														
Maltose eq− C2	0.14	1													
TSI	0.22	0.50**	1												
Cit	−0.15	0.21	−0.07	1											
Tar	−0.08	−0.05	−0.19	0.42**	1										
Mal	0.31**	0.23*	−0.03	0.53**	0.26*	1									
Fum	0.14	−0.04	−0.07	0.32**	0.65**	0.45**	1								
Prop	0.72**	0.01	0.15	0.06	−0.13	0.27*	0.11	1							
Shik	0.29*	0.05	0.27*	0.33**	0.54**	0.29*	0.66**	0.27*	1						
Formic eq−C3	0.07	−0.18	0.22	−0.05	0.33**	−0.01	0.47**	0.01	0.50**	1					
Oxalic eq−C4	−0.14	−0.17	−0.22	−0.13	0.23	0.06	0.04	−0.12	0.003	0.12	1				
CGA	−0.13	0.24*	0.02	0.40**	−0.33**	0.12	−0.27*	0.18	−0.12	−0.38**	−0.24*	1			
Cfa	−0.04	−0.05	−0.10	0.24*	0.33**	0.02	0.27*	0.08	0.32**	0.24*	0.14	0.19	1		
Kmp	−0.08	0.11	−0.05	0.44**	0.23	0.23	0.17	−0.10	0.20	0.05	−0.02	0.34**	0.35**	1	
GA	−0.07	0.06	0.08	0.33**	−0.38**	0.03	−0.45**	0.22	−0.22	−0.34**	−0.18	0.68**	0.09	0.19	1
pCQA	0.10	0.40**	0.30**	0.19	−0.36**	0.07	−0.40**	0.26*	−0.19	−0.39**	−0.45**	0.52**	−0.09	−0.11	0.51**
K3MalG	−0.24*	0.40**	0.33**	0.37**	−0.32**	−0.01	−0.38**	0.03	−0.10	−0.27*	−0.41**	0.62**	−0.08	0.13	0.57**
Q3Glu	−0.14	0.34**	0.35**	0.34**	−0.16	−0.003	−0.30*	0.05	−0.02	−0.22	−0.40**	0.54**	−0.04	0.11	0.48**
Q3MalGlc	−0.06	0.31**	0.29*	0.25*	−0.46**	−0.02	−0.46**	0.21	−0.20	−0.37**	−0.38**	0.71**	−0.05	0.07	0.66**
Q3AcGlc	−0.06	0.31**	0.26*	0.33**	−0.39**	−0.01	−0.42**	0.20	−0.15	−0.36**	−0.39**	0.73**	0.05	0.12	0.69**
Q3MGlc7G	−0.20	0.32**	0.33**	0.27*	−0.33**	−0.09	−0.39**	0.02	−0.10	−0.24*	−0.42**	0.59**	0.01	0.12	0.53**
Q3G	0.13	0.21	0.18	0.14	−0.50**	0.02	−0.42**	0.43**	−0.21	−0.47**	−0.27*	0.67**	0.003	−0.02	0.66**
CQA−Glc	−0.13	0.18	−0.11	0.54**	0.30*	0.24*	0.29*	0.05	0.23*	−0.07	−0.19	0.52**	0.48**	0.43**	0.23
Caf3G	−0.28*	0.16	0.07	0.44**	0.34**	0.05	0.16	−0.15	0.33**	0.19	0.05	0.31**	0.67**	0.37**	0.22
CafG	−0.09	0.05	−0.10	0.36**	−0.13	0.15	−0.02	0.18	0.04	−0.18	−0.01	0.75**	0.43**	0.34**	0.50**
Chic	−0.34**	−0.04	−0.32**	0.22	0.13	0.003	0.10	−0.31**	−0.02	0.06	0.07	0.36**	0.33**	0.30*	0.04
OT	0.05	0.06	0.20	−0.16	0.01	0.08	0.01	−0.08	−0.03	0.03	−0.01	0.07	−0.14	−0.02	0.02

	pCQA	K3MalG	Q3Glu	Q3MalGlc	Q3AcGlc	Q3MGlc7G	Q3G	CQA−Glc	Caf3G	CafG	Chic	OT
pCQA	1											
K3MalG	0.76**	1										
Q3Glu	0.80**	0.88**	1									
Q3MalGlc	0.86**	0.90**	0.88**	1								
Q3AcGlc	0.83**	0.88**	0.87**	0.97**	1							
Q3MGlc7G	0.78**	0.93**	0.92**	0.91**	0.91**	1						
Q3G	0.79**	0.68**	0.65**	0.89**	0.87**	0.70**	1					
CQA−Glc	0.02	0.18	0.19	0.15	0.28*	0.16	0.11	1				
Caf3G	0.12	0.30**	0.37**	0.20	0.28*	0.38**	−0.01	0.46**	1			
CafG	0.14	0.19	0.14	0.33**	0.40**	0.22	0.40**	0.61**	0.34**	1		
Chic	−0.29*	−0.13	−0.20	−0.19	−0.14	−0.18	−0.28*	0.58**	0.30*	0.51**	1	
OT	0.04	−0.03	0.09	0.01	−0.01	−0.03	−0.02	0.06	−0.12	−0.07	0.10	1

**. Correlation is significant at the 0.01 level (2−tailed).

*. Correlation is significant at the 0.05 level (2−tailed).

TAC: total antioxidant capacity, TPC: total phenolic content, TSS: total soluble solids, Chla: chlorophyll a, Chlb: chlorophyll b, Chl(a+b): chlorophyll (a+b), Total Car: total carotenoids, Lp: lactucopicrin, Lc: lactucin, DHLc: 11β,13-dihydrolactucin, Lp-ox: lactucopicrin-15-oxalate, dLc: 8-deoxylactucin, DHLc-glu: 11β,13-dihydrolactucin-15-glucoside, Lc-ox: lactucin-15-oxalate, DHLp-ox: 11β,13-dihydrolactucopicrin-15-oxalate, DHLp: 11β,13-dihydrolactucopicrin, STL: total lactones, Myo: myo-inositol, Glc: glucose, Fru: fructose, Suc: sucrose, C1: unidentified compound 1 (arabinose equivalents), C2: unidentified compound 2 (maltose equivalents), TSI: total sweetness index, Cit: citric acid, Tar: tartaric acid, Mal: malic acid, Fum: fumaric acid, Prop: propionic acid, Shik: shikimic acid, C3: unidentified compound 3 (formic acid equivalents), C4: unidentified compound 4 (oxalic acid equivalents), CGA: chlorogenic acid, Cfa: caffeic acid, Kmp: kaempferol, GA: gallic acid, pCQA: *p*-coumaroylquinic acid, K3MalG: kaempferol(6-O-malonyl) glucoside, Q3Glu: quercetin-3′-O-glucuronide, Q3MalGlc: quercetin-3-O-(6″-O-malonyl)-glucoside, Q3AcGlc: quercetin 3-O-(6″-acetyl-glucoside), Q3MGlc7G: quercetin 3-O-(6"-malonyl-glucoside) 7-O-glucoside, Q3G: quercetin-3-glucoside, CQA-Glc: caffeoylquinic acid glucoside, Caf3G: caffeic acid-3-glucoside, CafG: caffeic acid glucuronide, Chic: chicoric acid, OT: overall taste.
